# The relationship between obstructive sleep apnea and osteoarthritis: evidence from an observational and Mendelian randomization study

**DOI:** 10.3389/fneur.2024.1425327

**Published:** 2024-06-28

**Authors:** Zhe Yang, Tian Lv, Linna Jin, Xiaoheng Lv, Xiaoying Zhu, Xiaoling Wang, Lisan Zhang, Chenghan Tu, Shiqin Chen, Xiongwei Yan

**Affiliations:** ^1^Nursing Department, Sir Run Run Shaw Hospital, Zhejiang University School of Medicine, Hangzhou, China; ^2^Department of Neurology, Zhuji Affiliated Hospital of Wenzhou Medical University, Zhuji, China; ^3^Department of Neurology, Sir Run Run Shaw Hospital, Affiliated with School of Medicine, Zhejiang University, Hangzhou, China; ^4^Medical College, Shaoxing University, Shaoxing, China; ^5^Department of Neurology, Second People’s Hospital of Yuhuan, Yuhuan, China; ^6^Department of Orthopedic Surgery, Second People’s Hospital of Yuhuan, Yuhuan, China

**Keywords:** obstructive sleep apnea, osteoarthritis, Mendelian randomization, NHANES (National Health and Nutrition Examination Survey), BMI—body mass index

## Abstract

**Objectives:**

Obstructive sleep apnea (OSA) and osteoarthritis (OA) are common comorbidities that significantly impact individuals’ quality of life. However, the relationship between OSA and OA remains unclear. This study aims to explore the connection between OSA and OA and evaluate causality using Mendelian randomization (MR).

**Methods:**

A total of 12,454 participants from the National Health and Nutrition Examination Survey (2009–2012) were included. OSA participants were identified based on self-reported interviews. The association between OA and OSA was assessed through multivariable logistic regression analysis. A two-sample MR was employed to investigate the relationship between OSA and OA, specifically hip OA and knee OA, utilizing the inverse variance-weighted (IVW) approach.

**Results:**

Based on the observational study, individuals with OSA exhibited a higher risk of OA (OR = 1.67, 95% CI = 1.40–1.98). IVW demonstrated that the risk of OA (OR = 1.13, 95% CI: 1.05–1.21, *p* = 0.001), hip OA (OR = 1.11, 95% CI: 1.04–1.18, *p* = 0.002), and knee OA (OR = 1.08, 95% CI: 1.02–1.14, *p* = 0.005) was significantly associated with OSA. Reverse MR analyses indicated no effect of OA on OSA. Additionally, body mass index (BMI) was found to mediate 36.9% (95% CI, 4.64–73.2%, *p* = 0.026) of the OSA effects on OA risk.

**Conclusion:**

The cross-sectional observational analysis unveiled noteworthy associations between OSA and OA. Meanwhile, findings from the MR study provide support for a causal role.

## 1 Introduction

OSA is characterized by recurrent upper respiratory tract obstructions during sleep, leading to a reduction or cessation of airflow (1). A total of 936 million individuals are affected by OSA worldwide, with 425 million individuals experiencing moderate-to-severe cases (2). OSA patients endure chronic intermittent hypoxemia and metabolic disorders (3), often accompanied by inflammatory diseases (4). Studies indicate common risk factors associated with OSA and OA patients (5). OA, the most prevalent musculoskeletal disease, affects synovial joints, causing joint pain, decreased mobility, and a diminished quality of life (6). In 2020, the global prevalence of OA affecting approximately 7.6% of the population has been increasing in recent years (7, 8), making OA the fourth leading global cause of disability (9, 10). The medical burden associated with OA is steadily increasing worldwide (11).

Both OSA and OA significantly impact individuals’ quality of life. OSA disrupts sleep quality, leading to fragmentation, while OA induces pain and discomfort, particularly during the night. Current drug efficacy for both diseases is limited (5), necessitating urgent and effective treatment strategies. Exploring the correlation and potential mediator between OSA and OA provides valuable insights into disease mechanisms and symptom exacerbation. This understanding can enhance strategies for managing symptoms and finally improve outcomes for patients affected by both OSA and OA.

MR is an innovative epidemiological approach employing genetic variables as instrumental variables (IV) to assess causal effects on outcomes; this approach is less susceptible to biases from confounding factors and reverse causality (12).

This study integrates an observational investigation within the National Health and Nutrition Examination Survey (NHANES) with MR techniques to elucidate the causal relationship between OSA and OA.

## 2 Methods

### 2.1 Study population

**Figure 1:**
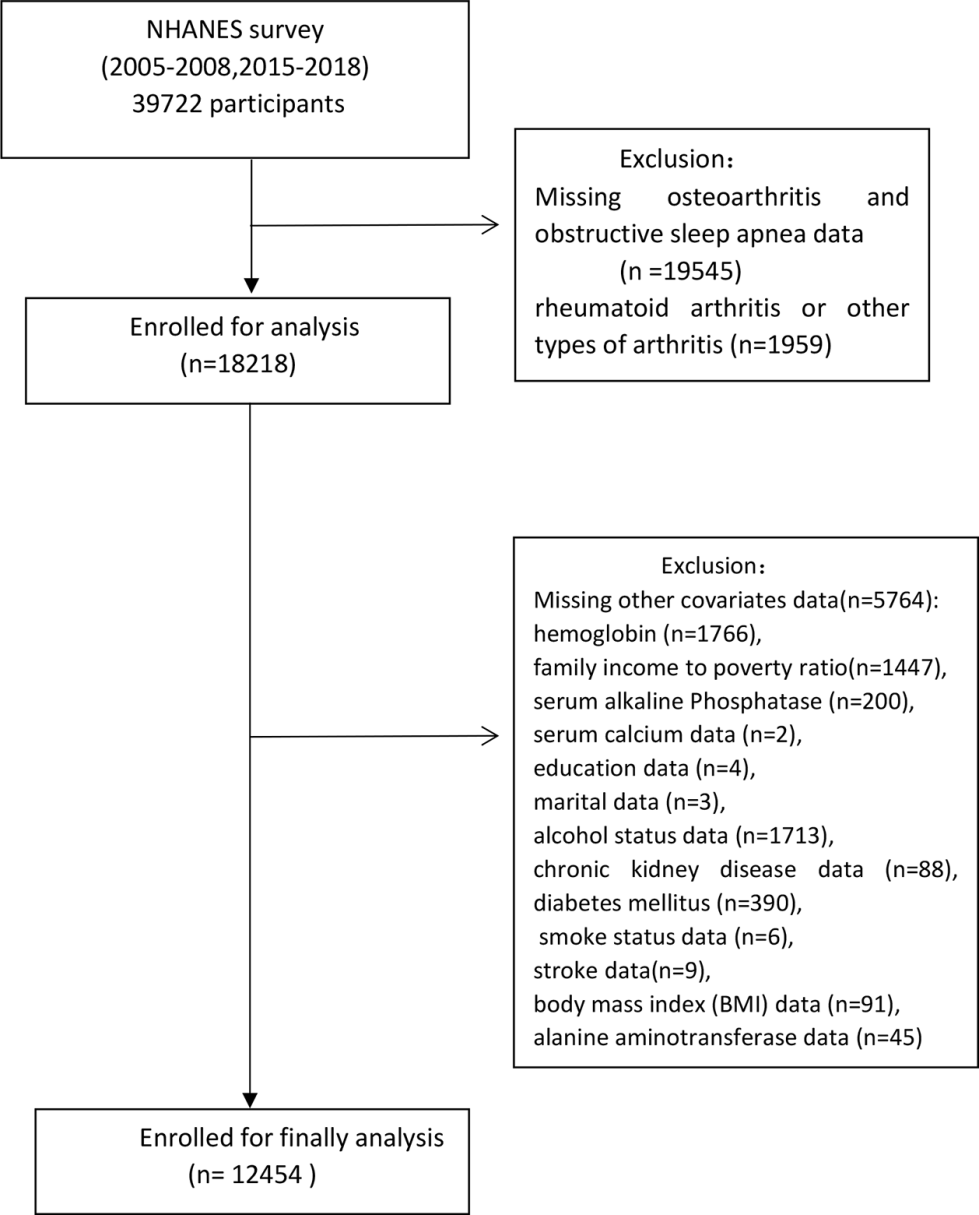
Flow chart of the study population.

The NHANES, a comprehensive research initiative evaluating the wellbeing and dietary status of individuals in the United States, comprises five core components: demographic details, dietary data, physical examinations, laboratory discoveries, and questionnaires. Ethical approval for NHANES protocols was duly granted by the National Center for Health Statistics Research Ethics Review Board, and all participants provided informed consent. This research included a total of 39,722 individuals from four NHANES cycles (2005–2006, 2007–2008, 2015–2016, and 2017–2018). The analysis excluded participants with missing OA data (*n* = 19,538) or OSA data (*n* = 7), as well as those with rheumatoid arthritis or other non-osteoarthritis forms (*n* = 1,959). Additionally, participants with missing covariate data were excluded, including family income-to-poverty ratio (PIR) data (*n* = 1,447), educational data (*n* = 4), marital status data (*n* = 3), smoking status data (*n* = 6), alcohol consumption data (*n* = 1,713), BMI data (*n* = 91), chronic kidney disease (CKD) data (*n* = 88), diabetes mellitus (DM) data (*n* = 390), stroke data (*n* = 9), hemoglobin (HB) data (*n* = 1,766), serum alkaline phosphatase (ALP) data (*n* = 200), serum calcium data (*n* = 2), and alanine aminotransferase (Alt) data (*n* = 45). Consequently, the analysis encompassed a total of 12,454 individuals, as illustrated in Figure 1 through a flow chart.

### 2.2 Variables

The exposure variable in this study was OSA, while the outcome variable was OA. OSA determination relied on affirmative responses to three yes-or-no questions related to snoring frequency, snorting or cessation of breathing occurrences, and daytime sleepiness. Individuals with positive responses to any of these questions were considered to display symptoms suggestive of OSA (13). OA participants in this study were identified based on self-reported personal interview data on various health conditions.

Demographics, medical conditions, and laboratory examinations were used to classify covariates. During home interviews, data on demographic characteristics, such as age, gender, marital status, educational attainment, PIR, and race, were gathered, along with information on medical conditions such as DM, hypertension, hyperlipidemia, and CKD. As a part of the NHANES laboratory examination, serum samples were collected, including serum calcium, Alt, ALP, creatinine, white blood cell count (WBC), and HB levels.

### 2.3 Mendelian randomization

For this research, we obtained genome-wide association study (GWAS) data on OSA from the Finnegan dataset population, which can be accessed at https://storage.googleapis.com/finngen-public-data-r9/summary_stats/finngen_R9_G6_SLEEPAPNO.gz. The study involved a total of 375,657 individuals, comprising 38,998 individuals with OSA and 336,659 individuals as controls. The GWAS catalog dataset (https://www.ebi.ac.uk/gwas/downloads/summary-statistics.ID GCST90044591) provided genetic data associated with OA from Jiang (14), consisting of 8,952 individuals diagnosed with OA and 447,396 control individuals without OA. The GWAS dataset for OA in the knees and hips was acquired from Tachmazidou et al. (15). BMI summary statistics were obtained from MRC-IEU, involving 461,460 samples (IEU GWAS ID ukb-b-19553). To mitigate population stratification bias, only studies including individuals of European descent were used to retrieve all summary data.

All GWAS studies included in this research received approval from the relevant ethical review boards, and participants provided written informed consent. The research adhered to the STROBE MR guideline (16).

### 2.4 Selection of instrumental variables

We selected instrumental variables (IVs) for OSA, OA, OA of the hip and knee, potential mediator [fasting insulin, Homeostasis Model Assessment of Insulin Resistance, Modified Stumvoll Insulin Sensitivity Index, and Modified Stumvoll Insulin Sensitivity Index (model adjusted for BMI)] GWAS data with a *p*-value of <5 × 10^−6^, ensuring independence (*r*^2^ < 0.001, kb = 10,000). The IVs for the potential mediator (BMI, waist circumference, hip circumference, waist-to-hip ratio adjusted for BMI, and waist-hip ratio) GWAS data were chosen based on a p-value of less than 5 × 10^−8^, ensuring independence (*r*^2^ < 0.001, kb = 10,000). The *F* statistic for each single nucleotide polymorphism (SNP) was computed using the formula Beta^2^/SE^2^.

### 2.5 Statistical analysis

This study meticulously incorporated intricate sampling designs and weights following the NHANES analytic guidelines, using mobile examination center (MEC) weights for all analyses. Continuous variables are presented as means and standard errors (SEs), while categorical variables are expressed as proportions. The examination of the relationship between OSA and OA involved a multivariate binary logistic regression model to calculate the odds ratio (OR) and 95% confidence intervals (CI). Three models were constructed for statistical inference. Model 1 solely included OSA, while Model 2 expanded to incorporate gender, age, ethnicity, marital status, and educational background. Model 3, an augmented version of Model 2, encompassed additional factors such as creatinine levels, alcohol consumption, BMI, smoking habits, serum calcium, serum Alt, ALP, serum creatinine, WBC, and medical history of hypertension, DM, CKD, stroke, and hyperlipidemia.

Subgroup analyses aimed to explore potential modifications in the impact of OSA on OA. These analyses considered age (<60, ≥60), sex, BMI (<30, ≥30), CKD status (yes, no), hyperlipidemia (yes, no), DM status (yes, no), hypertension (yes, no), and stroke status (yes, no), adjusting for Model 3.

**Figure 2:**
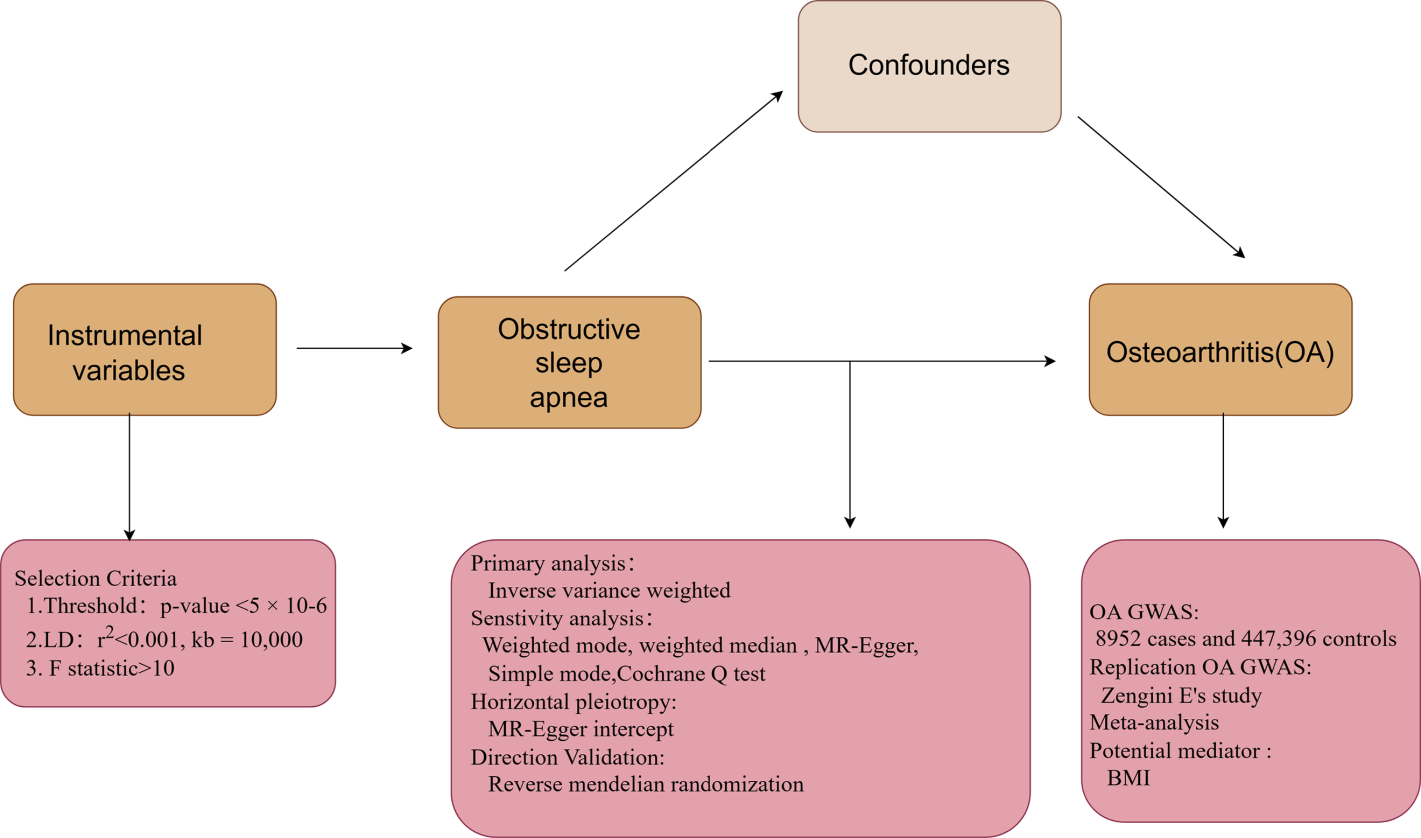
Flow chart of the Mendelian randomization analysis.

MR analyses included the computation of F statistics to gauge the strength of each instrument. An overview of the MR research design is displayed in Figure 2. The primary method, IVW, assessed the association of genetically predicted OSA and OA. Supplementary MR models, such as weighted mode, weighted median (WM), MR-Egger, and simple mode, were used. Cochrane *Q* test and MR-Egger intercept were used to examine potential heterogeneity and directional pleiotropy. A leave-one-out analysis identified significant single nucleotide polymorphisms (SNPs) and assessed the robustness of findings.

Furthermore, we used the IEU Open GWAS Project website^1^ to explore whether the genetic variants associated with OSA were also connected to other prevalent risk factors that might affect the results obtained from Mendelian randomization, including BMI, arthropathies, bone mineral density (BMD), vitamin D, and smoke (17, 18).

Reverse MR analyses, treating OA as the exposure and OSA as the outcome, were conducted to explore bidirectional causality, using the same GWAS datasets. The IVW examination was duplicated using an alternative osteoarthritis GWAS dataset from IEU GWAS, followed by a meta-analysis to consolidate outcomes.

To determine whether the observed association between OSA and OA was a direct association, we assessed the relationship between genetically previously established risk factors for OA (19) (BMI, waist circumference, hip circumference, waist-to-hip ratio adjusted for BMI, waist-hip ratio, fasting insulin, Homeostasis Model Assessment of Insulin Resistance, Modified Stumvoll Insulin Sensitivity Index, and Modified Stumvoll Insulin Sensitivity Index [model adjusted for BMI)] in MR analyses (GWAS dataset in Supplementary Table S1) (5). For significant associations, potential mediation effects (the exposure-mediator-outcome pathway) may exist. To explore the potential mediator between OSA and OA, a mediator MR analysis was conducted. This involved estimating the overall effect of OSA on OA (α), the effect of the potential mediator on OA (β2), and the effect of OSA on the potential mediator OA (β1). The direct impact of OSA on OA was calculated as α - β1*β2. Statistical analyses were conducted using R Studio 4.2.0 and the R package “Two Sample MR,” with a significance level set at a *p*-value of <0.05. Meta-analyses were carried out using RevMan 4.3.

## 3 Results

### 3.1 Observational study

#### 3.1.1 Baseline characteristics

The dataset under scrutiny included 12,454 participants. Table 1 presents the baseline characteristics categorized by OA. Of the participants, 1,560 (5.28%) had OA, and 3,628 (29.53%) had OSA. OSA participants exhibited a higher prevalence of OA (630, 39.84%) than those without OSA (2,998, 27.95%). The OA group was characterized by a higher likelihood of being male, former smokers, and having a history of DM, stroke, CKD, hyperlipidemia, hypertension, elevated serum creatinine, and BMI.

**Table 1:** Baseline characteristics of study participants based on the OA.

	Total	Non-OA	OA	*p*-value
Age	45.44 (0.32)	42.98 (0.288)	61.55 (0.45)	<0.0001
Sex				<0.0001
Female	6,101 (50.34)	5,099 (47.87)	1,002 (66.56)	
Male	6,337 (49.66)	5,782 (52.13)	555 (33.44)	
PIR	3.164 (0.04)	3.14 (0.05)	3.30 (0.08)	0.024
Serum ALP	68.81 (0.38)	68.20 (0.36)	72.81 (0.77)	<0.0001
Serum calcium	9.40 (0.01)	9.39 (0.01)	9.41 (0.02)	0.199
Serum ALT	25.43 (0.20)	25.68 (0.22)	23.84 (0.50)	0.001
HB	14.383 (0.04)	14.423 (0.04)	14.12 (0.05)	<0.0001
WBC	7.30 (0.04)	7.30 (0.04)	7.33 (0.07)	0.671
NLR	2.13 (0.02)	2.10 (0.02)	2.28 (0.04)	<0.0001
RWD	13.13 (0.02)	13.09 (0.02)	13.42 (0.05)	<0.0001
BMI	28.82 (0.13)	28.51 (0.13)	30.84 (0.24)	<0.0001
OSA				<0.0001
No	8,818 (70.49)	7,889 (72.06)	929 (60.18)	
Yes	3,620 (29.51)	2,992 (27.94)	628 (39.82)	
Education				0.486
College	6,805 (62.981)	5,918 (62.831)	887 (63.967)	
Non-college	5,633 (37.019)	4,963 (37.169)	670 (36.033)	
Marital status				0.001
Married	6,551 (55.89)	5,680 (55.03)	871 (61.51)	
Non-married	5,887 (44.11)	5,201 (44.97)	686 (38.49)	
Smoke				<0.0001
Former	2,862 (23.78)	2,306 (21.94)	556 (35.89)	
Never	7,039 (56.33)	6,281 (57.42)	758 (49.15)	
Now	2,537 (19.89)	2,294 (20.64)	243 (14.96)	
Stroke				<0.0001
No	12,074 (97.82)	10,644 (98.57)	1,430 (92.90)	
Yes	364 (2.18)	237 (1.43)	127 (7.10)	
DM				<0.0001
No	9,281 (79.36)	8,330 (81.42)	951 (65.86)	
IGT	457 (3.09)	392 (3.02)	65 (3.55)	
IFG	672 (5.46)	555 (5.08)	117 (7.88)	
DM	2,028 (12.10)	1,604 (10.49)	424 (22.71)	
CKD				<0.0001
No	10,483 (87.64)	9,362 (89.22)	1,121 (77.28)	
Yes	1,955 (12.36)	1,519 (10.78)	436 (22.72)	
Alcohol user				<0.0001
Never	1,714 (10.35)	1,496 (10.33)	218 (10.52)	
Former	1,719 (10.96)	1,415 (10.20)	304 (15.99)	
Heavy	2,676 (22.64)	2,516 (24.22)	160 (12.27)	
Mild	4,292 (37.51)	3,639 (36.30)	653 (45.48)	
Moderate	2,037 (18.53)	1,815 (18.96)	222 (15.74)	
Hyperlipidemia				<0.0001
No	3,832 (32.09)	3,549 (34.14)	283 (18.64)	
Yes	8,606 (67.91)	7,332 (65.86)	1,274 (81.36)	

Baseline characteristics of study participants. Mean ± SEs for continuous variables: *p*-value was calculated by weighted student’s *t*-test. Number (%) for categorical variables: *p*-value was calculated by weighted chi-square test. BMI, body mass index; DM, diabetes mellitus; IFG, impaired fasting glucose. IGT, impaired glucose tolerance; CKD, chronic kidney disease; RDW, red cell distribution width; HB, hemoglobin; PIR family income-to-poverty ratio; ALP, alkaline phosphatase; ALT, alanine aminotransferase; WBC, white blood cell; NLR, neutrophil-to-lymphocyte ratio; OSA, obstructive sleep apnea.

#### 3.1.2 Association between OSA and OA

Table 2 displays the outcomes of logistic regression analyses with multiple adjustments. After meticulous adjustments for various factors, Model 1 resulted in OR = 1.71, 95% CI = 1.46–2.00, and *p* < 0.0001; Model 2 showed OR = 1.98, 95% CI = 1.67–2.33, and *p* < 0.0001; and Model 3 revealed OR = 1.67, 95% CI = 1.40–1.99, and *p* < 0.0001.

**Table 2:** Independent associations between OSA and OA.

		Multivariable adjusted (OR, 95% CI)				
	Model 1		Model 2		Model 3	
	95% CI	*p*-value	95% CI	*p*-value	95% CI	*p*-value
OSA	1.71 (1.46, 2.00)	<0.0001	1.98 (1.68, 2.33)	<0.0001	1.67 (1.40, 1.99)	<0.0001

Model 1: OSA. Model 2: OSA, age, sex, education, and PIR. Model 3: OSA, age, sex, education, PIR, marriage status, HB, serum calcium, WBC, smoke, stroke, ALP, DM, hyperlipidemia, CKD, alcohol user, RDW, NLR, BMI, and ALT.

#### 3.1.3 Subgroup analyses

Subgroup analyses, considering variables such as age, sex, smoking status, history of CKD, hyperlipidemia, DM, hypertension, and stroke, consistently showed results with no significant interaction (all *p* interaction >0.05, Figure 1).

### 3.2 Mendelian randomization study

#### 3.2.1 MR analyses using primary genetic instruments

**Figure 3:**
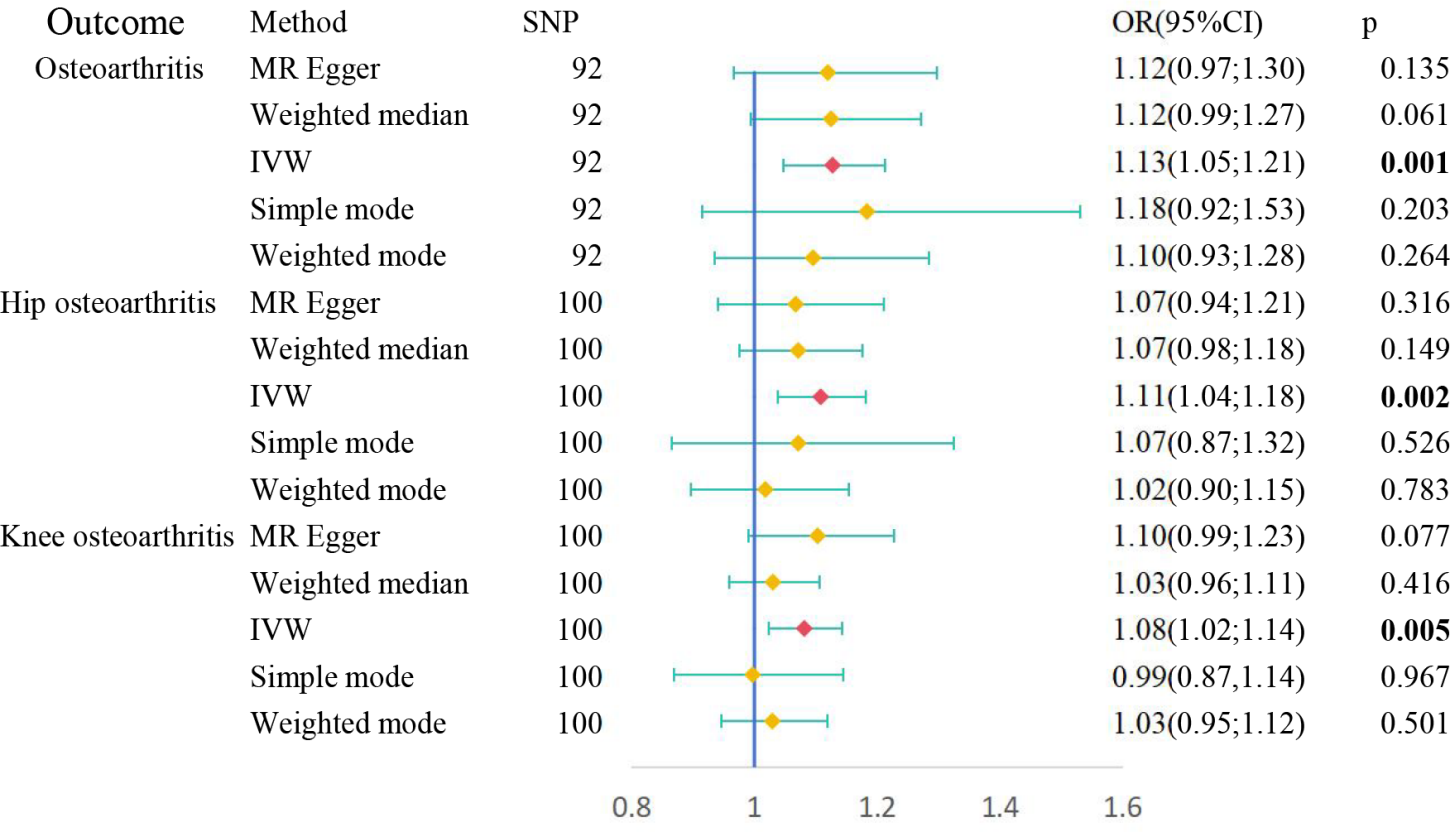
Causal relationships between OSA and OA risk performed by MR.

The genetic instrument for OSA (Supplementary Table S1) comprised 101 SNPs with *F* values exceeding 10 (see Supplementary Table S2). Employing the IVW method, the analysis indicated that OSA was associated with an increased likelihood of OA (OR = 1.13, 95% CI 1.05–1.21, *p* = 0.001), hip OA (OR = 1.11, 95% CI 1.04–1.18, *p* = 0.002), and knee OA (OR = 1.08, 95% CI 1.02–1.14, *p* = 0.005). Figure 3 visually represents outcomes from IVW, MR-Egger, weighted mode, weighted median, and simple mode.

**Figure 4:**
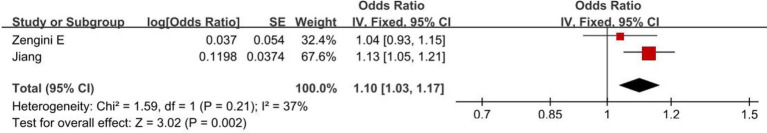
Meta-analyses on the relationship between OSA and OA.

The MR-Egger intercept test showed no horizontal pleiotropy regarding the impact of OSA on OA (intercept = 0.00046; *p* = 0.915), hip OA (intercept = 0.0038; *p* = 0.500), and knee OA (intercept = −0.00134; *p* = 0.861). The Cochrane Q test revealed no heterogeneity regarding the impact of OSA on OA (IVWQ 83.78, *p* = 0.691) but significant heterogeneity regarding OSA effects on hip OA (IVWQ 143.23, *p* = 0.00244) and knee OA (IVWQ 161.48, *p* = 7.45 10^−5^). The leave-one-out analysis found no SNP significantly influencing results (Figure 2–4).

After reviewing the IEU Open GWAS Project website, we discovered that 45 SNPs were linked to confounding factors [BMI (28), weight (3), obesity (1), waist circumference (1), arthropathies (6), BMD (2), vitamin D deficiency (2), and smoking (2)]. Then, we removed these 45 SNPs and found that the causality remained the same (OA IVW OR = 1.151, 95% CI: 1.042–1.273, *p* = 0.006, knee OA IVW OR = 1.064, 95% CI: 1.002–1.130, *p* = 0.04146917, hip OA IVW OR = 1.086, 95% CI: 1.002–1.178, *p* = 0.043).

#### 3.2.2 Bidirectional MR, replication, and meta-analysis

Reverse MR analyses indicated no evidence of a causal relationship between OSA and OA. Odds ratios (OR) for OA, knee OA, and hip OA were 1.00 [95%CI (0.97–1.03), *p* = 0.89], 1.04 (95% CI: 0.99–1.09, *p* = 0.08), and 0.98 (95% CI: 0.91–1.06, *p* = 0.67), respectively. Replication analysis used OA GWAS data from Zengini et al. (20) (IVW OR = 1.04, 95% CI = 0.93–1.15, *p* = 0.493), meta-analyses showing increased OA risk with a genetic predisposition for elevated OSA levels (OR = 1.10, 95% CI = 1.03–1.17, *p* = 0.002) (Figure 4).

#### 3.2.3 Mediator MR analyses

Given that waist circumference, hip circumference, BMI, and insulin resistance are well-established risk factors for OA, they could potentially mediate the effect of OSA on the risk of developing OA (Supplementary Figure S5 and Supplementary Table S5). Among the three potential mediators, we only identified BMI as a mediator between OSA and OA (Supplementary Table S3).

**Figure 5:**
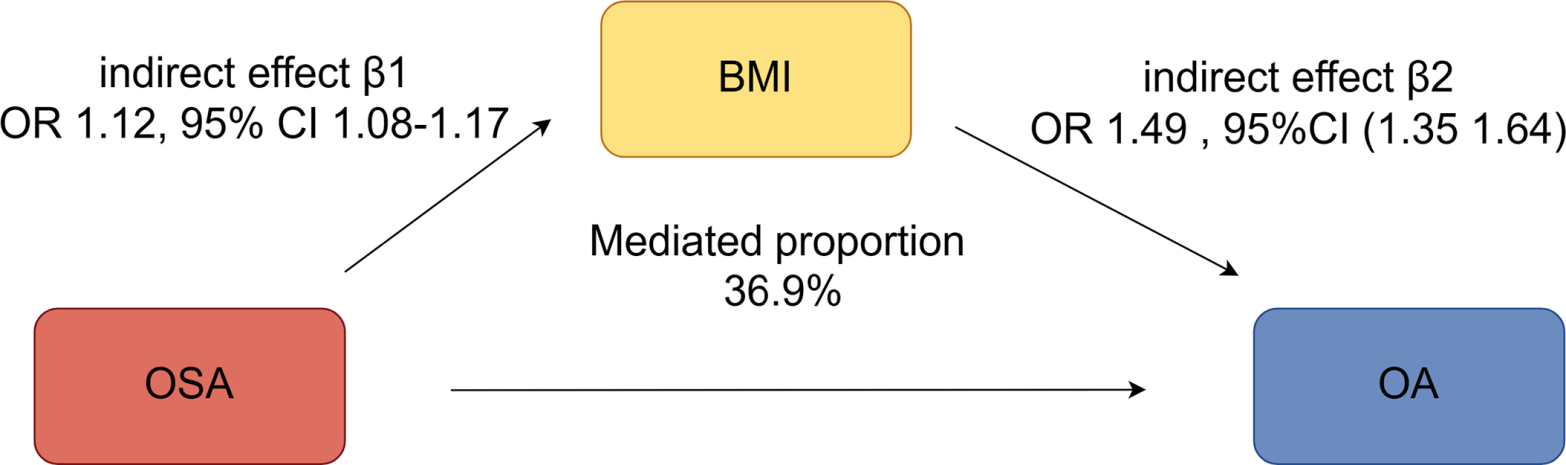
Mediation analysis of the effect of OSA on OA via BMI under a two-step Mendelian randomization analysis.

A total of 458 independent SNPs served as IVs for BMI, all with F statistics >10 (see Supplementary Table S4). Mediator MR analysis revealed that BMI (IVW: OR 1.49, 95% CI 1.35–1.64, *p* = 3.16e-15) was associated with an increased overall risk of OA (indirect effect β2). An increased BMI risk (indirect effect β1) was observed in relation to OSA (IVW OR 1.12, 95% CI 1.08–1.17, *p* = 2.46e-08). Furthermore, OSA demonstrated a causal association with heightened OA susceptibility (IVW OR = 1.13, 95% CI 1.05–1.21, *p* = 0.001) (overall effect α). The percentage of the impact of OSA on OA influenced by BMI was 36.9% (95% CI: 4.64–73.2%, *p* = 0.026) (Figure 5).

## 4 Discussion

This study represents the first comprehensive investigation into the relationship between OSA and OA through MR analysis, utilizing large-scale observational study data and genetic datasets. Cross-sectional observational analysis identified significant relationships between OSA and OA, while findings based on the MR study did support a causal role. The analysis of potential mediating factors found that BMI was an important factor between OSA and OA.

OSA and OA are common diseases. Previous studies found that the prevalence of OSA among individuals with OA was significantly higher than that in the general population, with figures standing at 66% versus 17%, respectively (21).

Sleep, as a period of physiological recovery, creates an environment conducive to cell proliferation, migration, and differentiation, with cell division and protein synthesis peaking during sleep (22, 23). OSA causes airway collapse, leading to oxygen deprivation and disruption of sleep rhythm (24–26). Previous studies revealed that OSA can cause increases in inflammatory, oxidative stress, and metabolic abnormalities, such as abnormal blood lipids, uric acid, and blood sugar (22, 23), which may lead to the future development of OA.

In this study, our MR analysis provided evidence of a causal relationship between OSA and OA, substantiated by rigorous examinations of horizontal pleiotropy and heterogeneity. These findings were further reinforced by two-way MR analysis, eliminating the possibility of reverse causality.

Obesity and inflammation are potential pathogenic mechanisms by which OSA leads to OA. Previous studies, through bioinformatics analysis, have found that shared inflammation genes between OSA and OA are significantly enriched in the TNF pathway and the IL-17 pathway (27).

The correlation between OSA and BMI has been a focal point of research for quite some time. The sleep fragmentation due to OSA can result in a state of effective sleep deprivation, which, in turn, can cause daytime drowsiness, a reduction in physical activity, and, consequently, an increase in body weight (28). One key factor contributing to weight gain in individuals with OSA is insulin resistance. Studies have indicated that the development of insulin resistance in the context of sustained intermittent hypoxia is closely tied to the disruption of leptin signaling pathways (28, 29).

In this study, we observed significant mediated effects of BMI on the associations between OSA and OA risk. In particular, other obesity and insulin resistance markers mediated no association between OSA and OA risk, among which the mediated proportion of BMI was 36.9%, consistent with previous research (30). These findings suggested that OSA may increase obesity and finally aggravate OA.

A high BMI or obesity leads to overloading the joints due to excess weight, which, in turn, leads to the destruction of articular cartilage (31). Others attributed it to excess fat tissue, which secretes hormones and proinflammatory cytokines, contributing to low-grade systemic inflammation (32). Given that BMI is a relatively controllable mediating factor (33), effectively managing BMI in OSA patients could potentially reduce the incidence of OA.

This research integrates a cross-sectional approach with Mendelian randomization, offering a foundational exploration to ascertain preliminary associations. Subsequently, the Mendelian randomization study reinforces these findings by substantiating the underlying causal mechanisms. Moreover, this methodology effectively mitigates the risk of false positives inherent in Mendelian studies, thereby enhancing the credibility of our results (34). The limitations of the study include the identification of OSA based on participants’ self-reported interviews. This methodology might have resulted in an inflated estimation of the true incidence of OSA. Furthermore, the absence of granular individual-level data from the GWAS precluded us from discerning whether the condition could introduce any inherent biases into our analysis. Finally, the applicability of our findings to diverse ethnic groups may be constrained, given that our analysis was focused on individuals of European descent, thus limiting the generalizability of our conclusions.

## 5 Conclusion

The study identified a causal relationship between OSA and OA and uncovered BMI as a mediator, laying a foundation for future research avenues and clinical interventions in the realm of sleep-related musculoskeletal disorders.

## Data availability statement

The raw data supporting the conclusions of this article will be made available by the authors, without undue reservation.

## Ethics statement

The studies involving humans were approved by NCHS Research Ethics Review Board. The studies were conducted in accordance with the local legislation and institutional requirements. Written informed consent for participation in this study was provided by the participants’ legal guardians/next of kin.

## Author contributions

ZY: Data curation, Methodology, Visualization, Writing – original draft, Writing – review & editing. TL: Data curation, Methodology, Visualization, Writing – original draft. LJ: Data curation, Supervision, Writing – original draft. XL: Data curation, Methodology, Supervision, Writing – original draft. XZ: Data curation, Methodology, Writing – original draft. XW: Data curation, Methodology, Writing – original draft. LZ: Data curation, Supervision, Validation, Visualization, Writing – original draft. CT: Data curation, Writing – original draft. SC: Data curation, Methodology, Writing – original draft. XY: Data curation, Methodology, Supervision, Writing – original draft, Writing – review & editing.

## Footnotes

^1^ http://gwas.mrcieu.ac.uk

## References

[1] Brodie KD Goldberg AN. Obstructive sleep apnea: a surgeon’s perspective. Med Clin North Am. (2021) 105:885–900. doi: 10.1016/j.mcna.2021.05.010 34391541

[2] Benjafield AV Ayas NT Eastwood PR Heinzer R Ip MSM Morrell MJ. Estimation of the global prevalence and burden of obstructive sleep apnoea: a literature-based analysis. Lancet Respir Med. (2019) 7:687–98. doi: 10.1016/S2213-2600(19)30198-5, PMID: 31300334 PMC7007763

[3] Giampá SQC Lorenzi-Filho G Drager LF. Obstructive sleep apnea and metabolic syndrome. Obesity. (2023) 31:900–11. doi: 10.1002/oby.23679 36863747

[4] Labarca G Vena D Hu WH Esmaeili N Gell L Yang HC. Sleep apnea physiological burdens and cardiovascular morbidity and mortality. Am J Respir Crit Care Med. (2023) 208:802–13. doi: 10.1164/rccm.202209-1808OC, PMID: 37418748 PMC10563185

[5] Gaspar LS Sousa C Álvaro AR Cavadas C Mendes AF. Common risk factors and therapeutic targets in obstructive sleep apnea and osteoarthritis: an unexpectable link? Pharmacol Res. (2021) 164:105369. doi: 10.1016/j.phrs.2020.105369, PMID: 33352231

[6] Hunter DJ Bierma-Zeinstra S. Osteoarthritis. Lancet. (2019) 393:1745–59. doi: 10.1016/S0140-6736(19)30417-9 31034380

[7] GBD 2021 Osteoarthritis Collaborators. Global, regional, and national burden of osteoarthritis, 1990–2020 and projections to 2050: a systematic analysis for the Global Burden of Disease Study 2021. Lancet Rheumatol. (2023) 5:e508–22. doi: 10.1016/S2665-9913(23)00163-7, PMID: 37675071 PMC10477960

[8] Turkiewicz A Petersson IF Björk J Hawker G Dahlberg LE Lohmander LS. Current and future impact of osteoarthritis on health care: a population-based study with projections to year 2032. Osteoarthr Cartil. (2014) 22:1826–32. doi: 10.1016/j.joca.2014.07.015, PMID: 25084132

[9] National Institute for Public Health and the Environment. Public health foresight study 2018 (VTV-2018): diseases. (2018). Available at: https://www.vtv2018.nl/en. (Accessed October 27, 2023).

[10] Woolf AD Pfleger B. Burden of major musculoskeletal conditions. Bull World Health Organ. (2003) 81:646–56. PMID: 14710506 PMC2572542

[11] Safiri S Kolahi AA Smith E Hill C Bettampadi D Mansournia MA. Global, regional and national burden of osteoarthritis 1990–2017: a systematic analysis of the Global Burden of Disease Study 2017. Ann Rheum Dis. (2020) 79:819–28. doi: 10.1136/annrheumdis-2019-216515, PMID: 32398285

[12] Kessler RC Bromet EJ. The epidemiology of depression across cultures. Annu Rev Public Health. (2013) 34:119–38. doi: 10.1146/annurev-publhealth-031912-114409, PMID: 23514317 PMC4100461

[13] Cavallino V Rankin E Popescu A Gopang M Hale L Meliker JR. Antimony and sleep health outcomes: NHANES 2009–2016. Sleep Health. (2022) 8:373–9. doi: 10.1016/j.sleh.2022.05.005, PMID: 35753957

[14] Jiang L Zheng Z Fang H Yang J. A generalized linear mixed model association tool for biobank-scale data. Nat Genet. (2021) 53:1616–21. doi: 10.1038/s41588-021-00954-4, PMID: 34737426

[15] Tachmazidou I Hatzikotoulas K Southam L Esparza-Gordillo J Haberland V Zheng J. Identification of new therapeutic targets for osteoarthritis through genome-wide analyses of UK Biobank data. Nat Genet. (2019) 51:230–6. doi: 10.1038/s41588-018-0327-1, PMID: 30664745 PMC6400267

[16] Skrivankova VW Richmond RC Woolf BAR Yarmolinsky J Davies NM Swanson SA. Strengthening the reporting of observational studies in epidemiology using Mendelian randomization: the STROBE-MR statement. JAMA. (2021) 326:1614–21. doi: 10.1001/jama.2021.18236, PMID: 34698778

[17] Zhu S Ji L He Z Zhang W Tong Y Luo J. Association of smoking and osteoarthritis in US (NHANES 1999–2018). Sci Rep. (2023) 13:3911. doi: 10.1038/s41598-023-30644-6, PMID: 36890196 PMC9995311

[18] Palazzo C Nguyen C Lefevre-Colau MM Rannou F Poiraudeau S. Risk factors and burden of osteoarthritis. Ann Phys Rehabil Med. (2016) 59:134–8. doi: 10.1016/j.rehab.2016.01.006 26904959

[19] Ho J Mak C Sharma V To K Khan W. Mendelian randomization studies of lifestyle-related risk factors for osteoarthritis: a PRISMA review and Meta-analysis. Int J Mol Sci. (2022) 23:11906. doi: 10.3390/ijms231911906, PMID: 36233208 PMC9570129

[20] Zengini E Hatzikotoulas K Tachmazidou I Steinberg J Hartwig FP Southam L. Genome-wide analyses using UK biobank data provide insights into the genetic architecture of osteoarthritis. Nat Genet. (2018) 50:549–58. doi: 10.1038/s41588-018-0079-y, PMID: 29559693 PMC5896734

[21] Taylor SS Hughes JM Coffman CJ Jeffreys AS Ulmer CS Oddone EZ. Prevalence of and characteristics associated with insomnia and obstructive sleep apnea among veterans with knee and hip osteoarthritis. BMC Musculoskelet Disord. (2018) 19:79. doi: 10.1186/s12891-018-1993-y, PMID: 29523117 PMC5845198

[22] Yang Z Lv T Lv X Wan F Zhou H Wang X. Association of serum uric acid with all-cause and cardiovascular mortality in obstructive sleep apnea. Sci Rep. (2023) 13:19606. doi: 10.1038/s41598-023-45508-2, PMID: 37949893 PMC10638300

[23] Kanbay A Köktürk O Pihtili A Ceylan E Tulu S Madenci E. Obstructive sleep apnea is a risk factor for osteoarthritis. Tuberk Toraks. (2018) 66:304–11. doi: 10.5578/tt.57403, PMID: 30683025

[24] Adamovich Y Ladeuix B Golik M Koeners MP Asher G. Rhythmic oxygen levels reset circadian clocks through HIF1α. Cell Metab. (2017) 25:93–101. doi: 10.1016/j.cmet.2016.09.014, PMID: 27773695

[25] Manella G Aviram R Bolshette N Muvkadi S Golik M Smith DF. Hypoxia induces a time- and tissue-specific response that elicits intertissue circadian clock misalignment. Proc Natl Acad Sci USA. (2020) 117:779–86. doi: 10.1073/pnas.1914112117, PMID: 31848250 PMC6955294

[26] Gabryelska A Turkiewicz S Karuga FF Sochal M Strzelecki D Białasiewicz P. Disruption of circadian rhythm genes in obstructive sleep apnea patients-possible mechanisms involved and clinical implication. Int J Mol Sci. (2022) 23:709. doi: 10.3390/ijms23020709, PMID: 35054894 PMC8775490

[27] Weng L Luo X Luo Y Zhang Q Yao K Tan J. Association between sleep apnea syndrome and osteoarthritis: insights from bidirectional Mendelian randomization and bioinformatics analysis. Nat Sci Sleep. (2024) 16:473–87. doi: 10.2147/NSS.S461010, PMID: 38737460 PMC11088414

[28] Polotsky VY Li J Punjabi NM Rubin AE Smith PL Schwartz AR. Intermittent hypoxia increases insulin resistance in genetically obese mice. J Physiol. (2003) 552:253–64. doi: 10.1113/jphysiol.2003.048173, PMID: 12878760 PMC2343324

[29] Pillar G Shehadeh N. Abdominal fat and sleep apnea: the chicken or the egg? Diabetes Care. (2008) 31:S303–9. doi: 10.2337/dc08-s272 18227501 PMC2453667

[30] Lei T Li M Qian H Yang J Hu Y Hua L. The effect of sleep on metabolism, musculoskeletal disease, and mortality in the general US population: analysis of results from the National Health and Nutrition Examination Survey. JMIR Public Health Surveill. (2023) 9:e46385. doi: 10.2196/46385, PMID: 37934562 PMC10664015

[31] Nedunchezhiyan U Varughese I Sun ARJ Wu X Crawford R Prasadam I. Obesity, inflammation, and immune system in osteoarthritis. Front Immunol. (2022) 13:907750. doi: 10.3389/fimmu.2022.907750, PMID: 35860250 PMC9289681

[32] Lerman SF Finan PH Smith MT Haythornthwaite JA. Psychological interventions that target sleep reduce pain catastrophizing in knee osteoarthritis. Pain. (2017) 158:2189–95. doi: 10.1097/j.pain.0000000000001023, PMID: 28767510 PMC5640483

[33] Vitiello MV McCurry SM Shortreed SM Baker LD Rybarczyk BD Keefe FJ. Short-term improvement in insomnia symptoms predicts long-term improvements in sleep, pain, and fatigue in older adults with comorbid osteoarthritis and insomnia. Pain. (2014) 155:1547–54. doi: 10.1016/j.pain.2014.04.032, PMID: 24793909 PMC4104256

[34] Wang M Jian Z Ma Y Jin X Li H Wang K. Depression increases the risk of kidney stone: results from the National Health and Nutrition Examination Survey 2007–2018 and Mendelian randomization analysis. J Affect Disord. (2022) 312:17–21. doi: 10.1016/j.jad.2022.06.008, PMID: 35691420

