# Supplementary material for: The relationship between obstructive sleep apnea and osteoarthritis: evidence from an observational and Mendelian randomization study
Source: Front Neurol. 2024 Jun 28;15:1425327. doi: 10.3389/fneur.2024.1425327 (PMC11239388; doi:10.3389/fneur.2024.1425327)
Supplement: Supplementary file 1 [file Data_Sheet_1.docx]

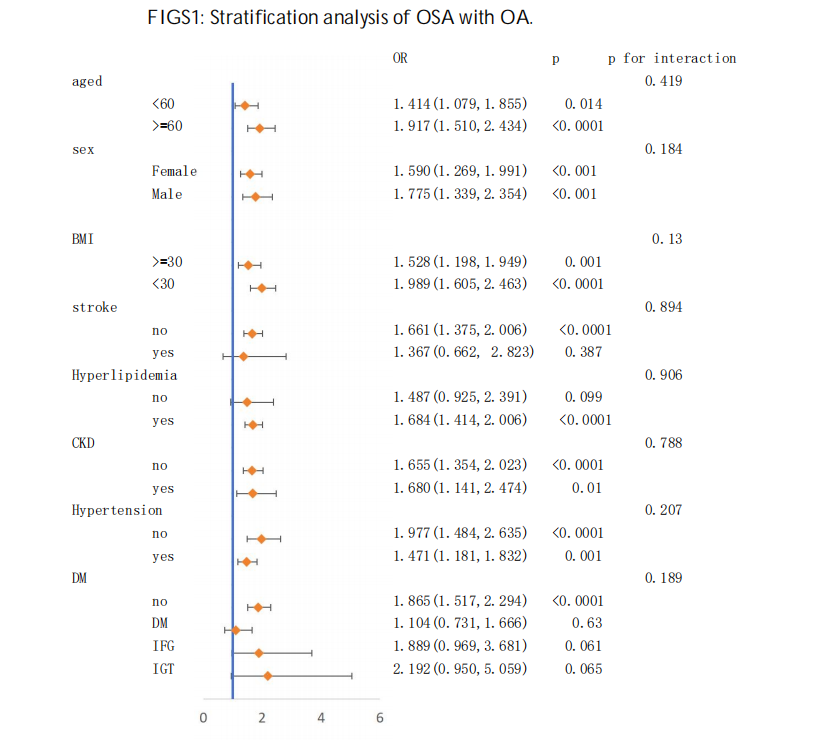


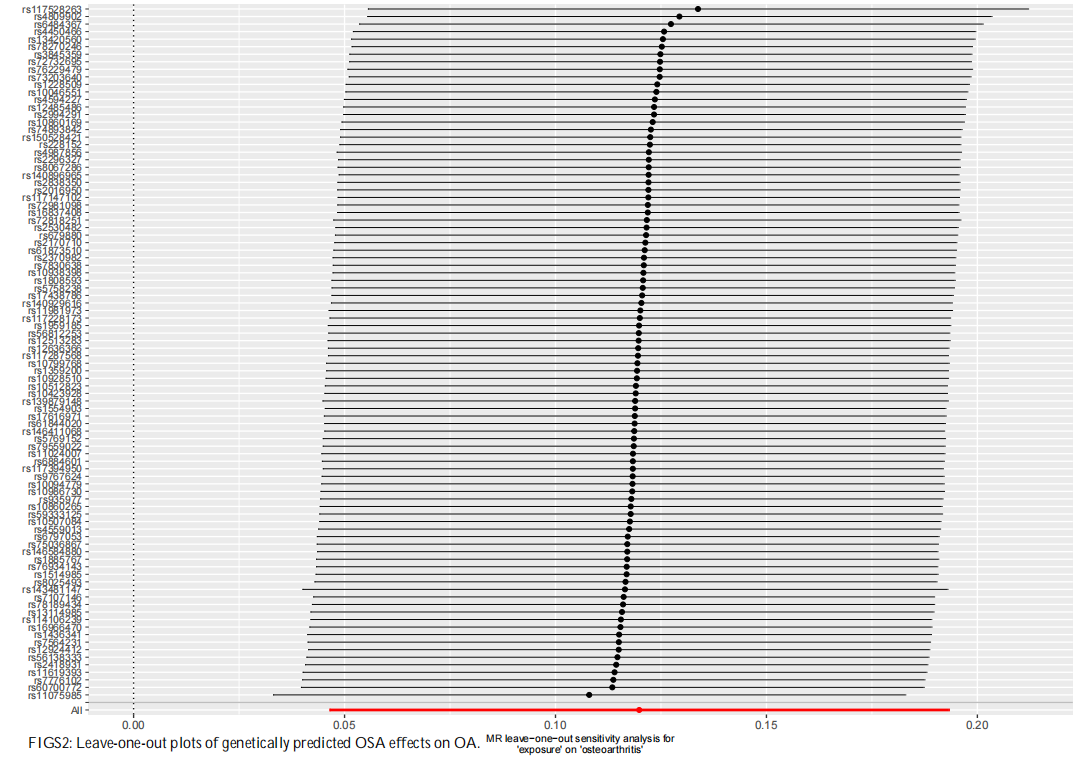


FIGS2 Leave-one-out plots of genetically predicted OSA effects on OA.


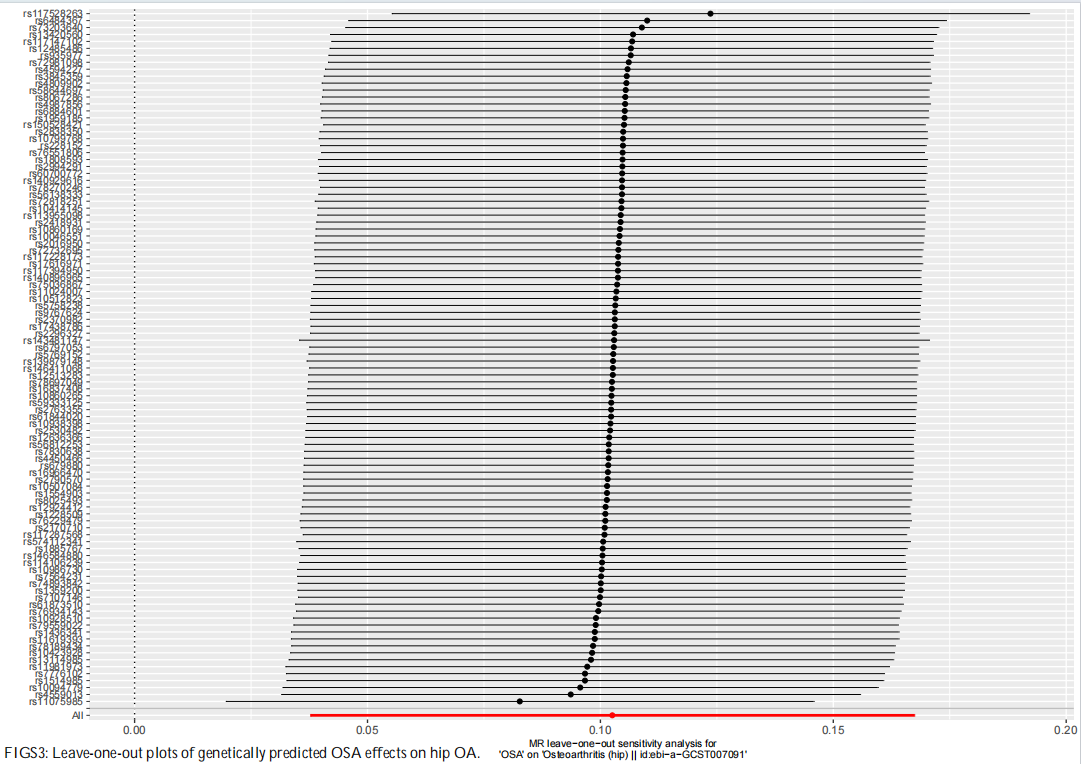


FIGS3 Leave-one-out plots of genetically predicted OSA effects on hip OA.


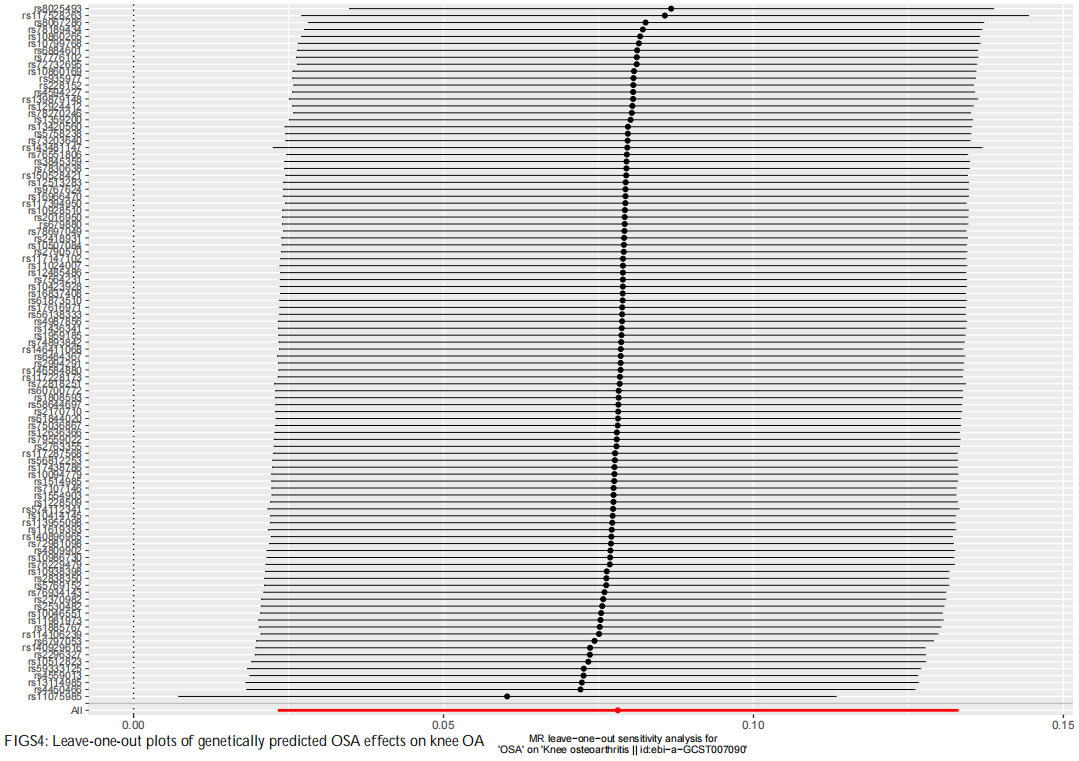


FIGS4 Leave-one-out plots of genetically predicted OSA effects on knee OA


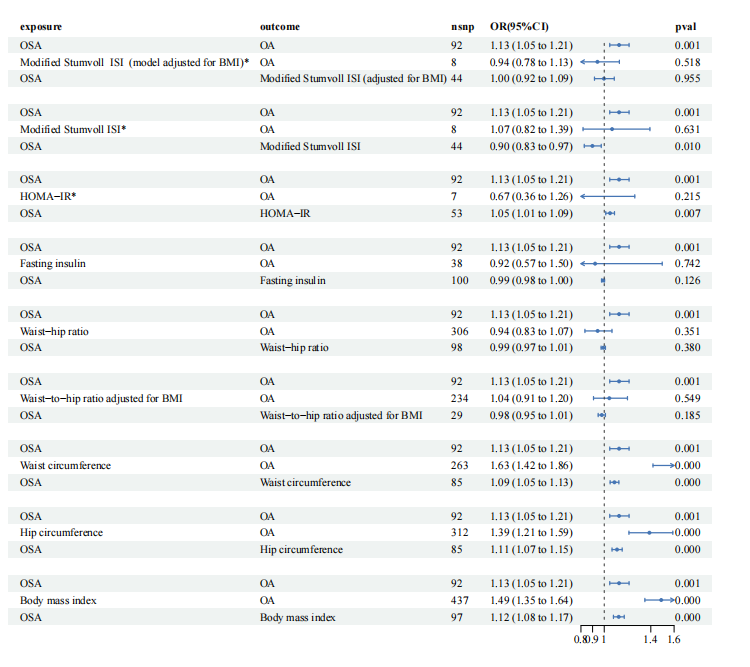


FIGS5 Forest plots of potential mediators underlying the effect of OSA on the OA risk
