# Supplementary material for: The relationship between obstructive sleep apnea and osteoarthritis: evidence from an observational and Mendelian randomization study
Source: Front Neurol. 2024 Jun 28;15:1425327. doi: 10.3389/fneur.2024.1425327 (PMC11239388; doi:10.3389/fneur.2024.1425327)
Supplement: Supplementary file 2 [file Table_1.docx]

| **Table S1. previously reported risk factors for OA** | | | |
| --- | --- | --- | --- |
| **Risk Factor** | **Consortium/Study** | **GWAS ID/PMID** | **sample size** |
| Body mass index(BMI) | MRC-IEU | ukb-b-19953 | 461,460 |
| Waist circumference | / | ebi-a-GCST90014020/34017140 | 407,661 |
| Waist-to-hip ratio adjusted for BMI | / | ebi-a-GCST90025996/34226706 | 458,349 |
| Waist-to-hip ratio | / | ebi-a-GCST90029009/29892013 | 502,773 |
| Hip circumference | / | ebi-a-GCST90014021/34017140 | 407,662 |
| HOMA-IR | / | ebi-a-GCST005179/20081858 | 37,037 |
| Fasting insulin | / | ebi-a-GCST90002238/34059833 | 151,013 |
| Modified Stumvoll ISI | / | ebi-a-GCST005178/27416945 | 16,753 |
| Modified Stumvoll ISI(adjusted for BMI) | / | ebi-a-GCST003658/27416945 | 16,753 |
| Abbreviations: HOMA-IR: Homeostasis model assessment of insulin resistance;Modified Stumvoll ISI: Modified Stumvoll Insulin Sensitivity Index | | | |
