# Supplementary material for: The relationship between obstructive sleep apnea and osteoarthritis: evidence from an observational and Mendelian randomization study
Source: Front Neurol. 2024 Jun 28;15:1425327. doi: 10.3389/fneur.2024.1425327 (PMC11239388; doi:10.3389/fneur.2024.1425327)
Supplement: Supplementary file 3 [file Table_2.pdf]

TableS2:Provides SNP from OSA GWAS details in the supplement.

exposure	SNP	effect_allele	other_allele	eaf	beta	se	pval	F
OSA	rs17438786	C	G	0.038	0.091	0.020	4.23E-06	21.157
OSA	rs10799768	T	G	0.172	-0.052	0.010	3.49E-07	25.958
OSA	rs140929616	C	T	0.023	0.126	0.025	6.14E-07	24.867
OSA	rs3845359	T	C	0.559	-0.038	0.008	8.26E-07	24.297
OSA	rs1228509	C	A	0.656	0.047	0.008	6.41E-09	33.706
OSA	rs16837408	T	C	0.228	-0.046	0.009	8.75E-07	24.186
OSA	rs1554903	C	G	0.106	0.062	0.012	6.86E-07	24.655
OSA	rs13420560	T	C	0.060	0.076	0.016	1.85E-06	22.750
OSA	rs76229479	C	A	0.099	-0.078	0.013	2.64E-09	35.436
OSA	rs10928510	G	A	0.160	-0.058	0.011	5.46E-08	29.545
OSA	rs7564231	G	A	0.880	0.060	0.012	6.58E-07	24.734
OSA	rs12485486	G	C	0.341	0.039	0.008	1.52E-06	23.117
OSA	rs1436341	A	G	0.881	0.065	0.012	6.83E-08	29.112
OSA	rs10512823	C	T	0.078	-0.073	0.015	5.43E-07	25.104
OSA	rs114106239	T	C	0.037	-0.121	0.021	1.19E-08	32.508
OSA	rs6797053	C	T	0.641	-0.037	0.008	4.79E-06	20.919
OSA	rs12636366	T	C	0.057	-0.092	0.017	5.43E-08	29.557
OSA	rs935977	A	G	0.367	-0.041	0.008	3.64E-07	25.877
OSA	rs1514985	A	G	0.475	0.037	0.008	1.05E-06	23.841
OSA	rs139879148	C	G	0.002	0.397	0.074	8.94E-08	28.591
OSA	rs10938398	A	G	0.472	0.035	0.008	4.08E-06	21.229
OSA	rs13114985	G	T	0.338	0.046	0.008	2.01E-08	31.484
OSA	rs12513283	G	T	0.283	0.040	0.008	3.18E-06	21.705
OSA	rs2170710	C	T	0.076	0.076	0.014	1.14E-07	28.115
OSA	rs76934143	T	A	0.046	0.083	0.018	4.35E-06	21.105
OSA	rs6884601	C	A	0.441	-0.037	0.008	1.68E-06	22.933
OSA	rs72818251	T	C	0.026	-0.117	0.025	2.96E-06	21.841
OSA	rs4559013	G	A	0.552	-0.037	0.008	1.98E-06	22.610
OSA	rs60700772	C	T	0.221	0.052	0.009	2.20E-08	31.312
OSA	rs228152	C	T	0.159	0.052	0.010	7.46E-07	24.492
OSA	rs7776102	T	G	0.250	-0.043	0.009	1.17E-06	23.633
OSA	rs9767624	C	T	0.628	-0.038	0.008	1.85E-06	22.741
OSA	rs76551806	T	C	0.068	0.072	0.015	2.11E-06	22.492
OSA	rs2994291	G	T	0.705	0.039	0.008	4.68E-06	20.964
OSA	rs117287568	C	A	0.038	-0.095	0.021	4.76E-06	20.931
OSA	rs117394950	G	A	0.036	-0.099	0.021	2.71E-06	22.013
OSA	rs10046551	C	G	0.744	-0.041	0.009	3.47E-06	21.539
OSA	rs11981973	G	A	0.182	0.061	0.010	4.72E-10	38.789
OSA	rs2530482	A	C	0.739	-0.046	0.009	2.65E-07	26.487
OSA	rs146584880	A	G	0.021	0.128	0.026	1.27E-06	23.474
OSA	rs1808593	T	G	0.838	-0.052	0.010	4.20E-07	25.602
OSA	rs10094779	G	A	0.308	0.045	0.008	5.31E-08	29.599
OSA	rs74893842	G	A	0.111	-0.062	0.012	5.01E-07	25.260
OSA	rs117528263	A	G	0.001	0.589	0.122	1.41E-06	23.272
OSA	rs7830638	G	A	0.190	0.047	0.010	1.52E-06	23.126
OSA	rs56138333	A	G	0.069	-0.074	0.015	1.95E-06	22.643
OSA	rs143481147	A	G	0.001	0.676	0.147	4.02E-06	21.255
OSA	rs679880	A	G	0.745	0.050	0.009	2.53E-08	31.040
OSA	rs78270246	T	A	0.078	0.067	0.014	3.29E-06	21.642
OSA	rs79559022	T	G	0.052	0.079	0.017	3.00E-06	21.814
OSA	rs72732695	T	C	0.028	0.118	0.023	1.76E-07	27.280
OSA	rs78189434	G	C	0.101	0.064	0.013	4.37E-07	25.524
OSA	rs10986730	T	C	0.524	-0.047	0.008	1.16E-09	37.029
OSA	rs574112341	A	G	0.002	0.413	0.083	5.74E-07	24.999
OSA	rs61844020	T	C	0.068	-0.075	0.016	1.66E-06	22.951
OSA	rs113955098	A	G	0.067	-0.099	0.016	3.38E-10	39.444
OSA	rs75036867	T	C	0.137	-0.054	0.011	1.68E-06	22.931
OSA	rs146411068	C	G	0.012	0.158	0.034	3.23E-06	21.676
OSA	rs2763355	A	G	0.497	0.036	0.008	4.58E-06	21.007
OSA	rs61873510	T	G	0.301	0.046	0.008	3.28E-08	30.534
OSA	rs117147102	A	G	0.022	0.122	0.026	1.96E-06	22.638
OSA	rs11024007	T	A	0.133	0.056	0.011	6.70E-07	24.698

OSA	rs7107146	T	C	0.108	0.058	0.012	2.14E-06	22.467
OSA	rs6484367	A	G	0.503	0.047	0.008	1.13E-09	37.087
OSA	rs2418931	T	C	0.310	0.041	0.008	8.72E-07	24.193
OSA	rs59333125	C	A	0.081	-0.082	0.014	1.32E-08	32.299
OSA	rs10860169	G	A	0.289	-0.041	0.009	1.78E-06	22.821
OSA	rs10507084	T	C	0.179	0.065	0.010	8.23E-11	42.203
OSA	rs117228173	C	T	0.054	-0.086	0.017	7.85E-07	24.394
OSA	rs10860265	T	C	0.431	0.036	0.008	2.46E-06	22.199
OSA	rs2016950	T	C	0.158	-0.059	0.011	4.13E-08	30.085
OSA	rs11619393	C	T	0.077	0.068	0.014	1.71E-06	22.902
OSA	rs1885767	G	A	0.594	-0.040	0.008	3.67E-07	25.861
OSA	rs73203640	T	G	0.210	0.043	0.009	3.40E-06	21.577
OSA	rs1359200	G	A	0.480	-0.036	0.008	4.11E-06	21.213
OSA	rs56812253	C	T	0.227	-0.045	0.009	1.22E-06	23.552
OSA	rs150528421	G	A	0.034	0.096	0.021	3.86E-06	21.335
OSA	rs2790570	G	A	0.580	-0.038	0.008	1.15E-06	23.656
OSA	rs2296327	T	C	0.292	0.040	0.008	2.99E-06	21.824
OSA	rs2370982	T	C	0.238	0.052	0.009	7.97E-09	33.283
OSA	rs1959185	A	G	0.135	0.059	0.011	1.19E-07	28.031
OSA	rs78697049	T	A	0.048	0.082	0.018	4.10E-06	21.216
OSA	rs17616971	A	G	0.565	0.036	0.008	3.48E-06	21.532
OSA	rs8025493	A	G	0.560	-0.038	0.008	7.00E-07	24.616
OSA	rs4594227	G	A	0.418	0.037	0.008	2.45E-06	22.203
OSA	rs11075985	A	C	0.429	0.082	0.008	2.15E-26	######
OSA	rs12924412	C	T	0.142	0.055	0.011	3.91E-07	25.738
OSA	rs13333522	G	C	0.527	0.042	0.008	4.06E-08	30.119
OSA	rs8067286	G	A	0.523	0.039	0.008	5.79E-07	24.979
OSA	rs4450466	T	C	0.122	0.059	0.012	4.16E-07	25.618
OSA	rs4987856	T	C	0.050	-0.087	0.018	1.73E-06	22.868
OSA	rs10414145	G	C	0.131	-0.055	0.012	2.71E-06	22.013
OSA	rs72981098	G	A	0.138	0.056	0.011	4.98E-07	25.270
OSA	rs16966470	G	C	0.077	-0.070	0.015	1.57E-06	23.057
OSA	rs10423928	A	T	0.259	-0.051	0.009	7.55E-09	33.389
OSA	rs58644697	A	G	0.230	-0.044	0.009	1.63E-06	22.983
OSA	rs4809902	C	G	0.228	-0.056	0.009	1.51E-09	36.516
OSA	rs140896965	T	C	0.049	-0.113	0.018	1.11E-09	37.130
OSA	rs2838350	C	T	0.677	0.038	0.008	4.34E-06	21.110
OSA	rs5758238	A	G	0.609	0.041	0.008	1.43E-07	27.680
OSA	rs5769152	T	G	0.556	0.040	0.008	3.08E-07	26.198
