# Supplementary material for: The relationship between obstructive sleep apnea and osteoarthritis: evidence from an observational and Mendelian randomization study
Source: Front Neurol. 2024 Jun 28;15:1425327. doi: 10.3389/fneur.2024.1425327 (PMC11239388; doi:10.3389/fneur.2024.1425327)
Supplement: Supplementary file 4 [file Table_3.docx]

TableS3: result of potential mediators on OSA and OA

| Exposure | Mediator | Outcome | indirect effect(95%CI) | P |
| --- | --- | --- | --- | --- |
| OSA | Body mass index | OA | 36.9%(4.64%–73.2%) | 0.026 |
| OSA | Hip circumference | OA | 28.5%(-10.3%–67.3%) | 0.151 |
| OSA | Waist circumference | OA | 34.0%(-21.9%–89.9%) | 0.234 |
