# Supplementary material for: The relationship between obstructive sleep apnea and osteoarthritis: evidence from an observational and Mendelian randomization study
Source: Front Neurol. 2024 Jun 28;15:1425327. doi: 10.3389/fneur.2024.1425327 (PMC11239388; doi:10.3389/fneur.2024.1425327)
Supplement: Supplementary file 5 [file Table_4.pdf]

TableS4: Provides SNP from BMI GWAS details in the supplement.

exposure	SNP	pos	pval	chr	beta	se	effect_allele	other_allele	eaf	F
Body mass index (BMI)	rs1000096	38692835	5.10E-11	4	-0.013	0.002	T	C	0.401	43.130
Body mass index (BMI)	rs10063055	140990108	1.70E-09	5	0.014	0.002	T	C	0.253	36.307
Body mass index (BMI)	rs10099330	143383694	4.20E-10	8	0.012	0.002	G	A	0.453	39.019
Body mass index (BMI)	rs10144067	93885198	5.60E-20	14	0.019	0.002	T	C	0.591	83.766
Body mass index (BMI)	rs10160769	76474827	1.20E-10	11	-0.016	0.002	C	G	0.218	41.413
Body mass index (BMI)	rs10169594	41637688	3.00E-09	2	0.012	0.002	C	T	0.363	35.171
Body mass index (BMI)	rs10172070	208307761	6.70E-09	2	0.016	0.003	T	C	0.146	33.618
Body mass index (BMI)	rs10182416	104242992	3.70E-11	2	0.013	0.002	G	A	0.512	43.741
Body mass index (BMI)	rs10423928	46182304	3.40E-42	19	-0.034	0.002	A	T	0.194	185.302
Body mass index (BMI)	rs10505836	19288508	1.20E-10	12	0.018	0.003	C	A	0.860	41.459
Body mass index (BMI)	rs10510025	118650996	2.20E-14	10	0.018	0.002	T	C	0.247	58.376
Body mass index (BMI)	rs1064213	198950240	3.70E-14	2	0.015	0.002	A	G	0.478	57.323
Body mass index (BMI)	rs10742752	45438374	6.10E-09	11	0.012	0.002	C	T	0.612	33.790
Body mass index (BMI)	rs10756714	15885041	1.90E-25	9	-0.021	0.002	G	A	0.444	108.659
Body mass index (BMI)	rs10756792	16726119	4.90E-17	9	-0.019	0.002	T	C	0.743	70.363
Body mass index (BMI)	rs10760277	126093999	1.00E-11	9	0.014	0.002	T	C	0.385	46.329
Body mass index (BMI)	rs10780248	81370555	1.20E-09	9	-0.012	0.002	A	G	0.559	36.959
Body mass index (BMI)	rs1078141	142619393	4.90E-12	8	0.014	0.002	T	C	0.384	47.740
Body mass index (BMI)	rs10799778	23313353	6.00E-12	1	-0.018	0.003	G	T	0.834	47.336
Body mass index (BMI)	rs10809621	11859607	1.40E-09	9	-0.013	0.002	G	C	0.350	36.628
Body mass index (BMI)	rs10824211	76363107	4.30E-13	10	0.021	0.003	T	C	0.139	52.502
Body mass index (BMI)	rs10832778	17394073	1.40E-08	11	0.012	0.002	G	C	0.623	32.131
Body mass index (BMI)	rs10887578	88096047	2.10E-11	10	0.013	0.002	C	G	0.497	44.852
Body mass index (BMI)	rs10927006	243557659	2.30E-09	1	-0.017	0.003	C	T	0.144	35.692
Body mass index (BMI)	rs10965698	23203619	3.90E-08	9	-0.011	0.002	T	C	0.370	30.186
Body mass index (BMI)	rs10989067	103119634	1.40E-15	9	0.017	0.002	A	G	0.316	63.725
Body mass index (BMI)	rs11001963	78760959	7.30E-09	10	0.012	0.002	T	C	0.581	33.456
Body mass index (BMI)	rs11009685	34511990	1.40E-08	10	-0.013	0.002	T	C	0.244	32.144
Body mass index (BMI)	rs11012732	21830104	7.10E-25	10	0.022	0.002	G	A	0.332	106.085
Body mass index (BMI)	rs11079849	47090785	1.80E-21	17	-0.020	0.002	T	C	0.329	90.543
Body mass index (BMI)	rs11099020	130724902	5.60E-12	4	-0.014	0.002	T	C	0.641	47.449
Body mass index (BMI)	rs11115160	82424100	2.20E-08	12	-0.013	0.002	A	G	0.238	31.326
Body mass index (BMI)	rs11122450	230301811	9.10E-09	1	-0.012	0.002	G	T	0.612	33.018
Body mass index (BMI)	rs11134679	170623391	1.20E-17	5	0.018	0.002	G	A	0.685	73.182
Body mass index (BMI)	rs11150745	78757626	2.90E-23	17	-0.021	0.002	G	A	0.318	98.736
Body mass index (BMI)	rs111598585	171635471	5.40E-09	4	-0.014	0.002	T	C	0.209	34.042
Body mass index (BMI)	rs11165643	96924097	4.90E-22	1	0.019	0.002	T	C	0.590	93.117
Body mass index (BMI)	rs111689389	27175962	4.70E-10	5	-0.014	0.002	C	G	0.283	38.781
Body mass index (BMI)	rs11218510	121922587	8.40E-13	11	-0.014	0.002	A	G	0.400	51.193
Body mass index (BMI)	rs11250094	10802001	2.30E-24	8	-0.020	0.002	C	G	0.548	103.747
Body mass index (BMI)	rs1126930	49399132	1.70E-09	12	0.032	0.005	C	G	0.035	36.260
Body mass index (BMI)	rs113079574	147354089	5.80E-10	4	-0.016	0.003	T	C	0.193	38.393
Body mass index (BMI)	rs113603865	39564930	1.80E-14	1	0.019	0.002	T	C	0.212	58.747
Body mass index (BMI)	rs113624107	88326386	2.10E-10	14	0.015	0.002	A	G	0.226	40.346
Body mass index (BMI)	rs115056380	111338672	2.50E-08	1	-0.026	0.005	A	G	0.048	31.069
Body mass index (BMI)	rs11525873	138817193	6.60E-13	7	-0.024	0.003	C	T	0.098	51.662
Body mass index (BMI)	rs11607476	115037061	3.10E-15	11	0.016	0.002	C	A	0.487	62.227
Body mass index (BMI)	rs11610621	121671133	3.10E-09	12	0.016	0.003	A	T	0.148	35.091
Body mass index (BMI)	rs11630647	99240947	2.70E-08	15	-0.013	0.002	A	G	0.252	30.878
Body mass index (BMI)	rs11634851	81028965	2.20E-09	15	0.012	0.002	G	C	0.465	35.757
Body mass index (BMI)	rs116374395	50723410	2.80E-09	5	0.032	0.005	A	G	0.035	35.349
Body mass index (BMI)	rs11641179	6716711	3.40E-08	16	-0.013	0.002	G	A	0.257	30.449
Body mass index (BMI)	rs11642090	81730582	2.90E-08	16	0.011	0.002	C	T	0.374	30.746
Body mass index (BMI)	rs11656076	31464270	7.60E-11	17	-0.015	0.002	A	G	0.225	42.360
Body mass index (BMI)	rs1167311	49996959	1.60E-19	1	-0.019	0.002	A	G	0.681	81.720
Body mass index (BMI)	rs11675464	204053742	1.60E-09	2	0.012	0.002	G	A	0.563	36.373
Body mass index (BMI)	rs11691869	100805996	5.60E-21	2	-0.019	0.002	A	C	0.362	88.296
Body mass index (BMI)	rs11699828	62157198	8.60E-09	20	-0.034	0.006	A	G	0.036	33.137
Body mass index (BMI)	rs11709402	131551027	4.90E-25	3	0.023	0.002	G	A	0.279	106.812
Body mass index (BMI)	rs117118217	131783328	1.30E-08	10	0.045	0.008	C	G	0.018	32.349
Body mass index (BMI)	rs117342986	54267868	1.60E-08	16	0.037	0.006	T	C	0.026	31.986
Body mass index (BMI)	rs11757278	13180454	9.90E-12	6	-0.015	0.002	C	T	0.304	46.355
Body mass index (BMI)	rs11778219	87762607	4.20E-09	8	0.016	0.003	G	A	0.163	34.549
Body mass index (BMI)	rs118136827	2168104	1.70E-09	17	-0.013	0.002	T	G	0.281	36.256
Body mass index (BMI)	rs11842871	31042452	2.80E-11	13	-0.015	0.002	T	G	0.260	44.299
Body mass index (BMI)	rs11919665	48085349	1.40E-09	3	-0.013	0.002	T	A	0.680	36.615
Body mass index (BMI)	rs12001437	34074476	2.80E-09	9	0.012	0.002	C	T	0.368	35.298
Body mass index (BMI)	rs12072739	98315893	3.30E-11	1	0.016	0.002	G	A	0.224	44.003

Body mass index (BMI)	rs12088284	80798635	8.90E-11	1	0.014	0.002	T	C	0.301	42.046
Body mass index (BMI)	rs12089815	91189933	5.70E-10	1	-0.012	0.002	A	G	0.549	38.431
Body mass index (BMI)	rs12140153	62579891	1.20E-21	1	-0.033	0.003	T	G	0.094	91.437
Body mass index (BMI)	rs12149660	70309237	3.00E-13	16	-0.023	0.003	A	G	0.115	53.234
Body mass index (BMI)	rs12259464	53680099	4.50E-11	10	0.013	0.002	A	G	0.484	43.402
Body mass index (BMI)	rs12273545	116911012	6.80E-09	11	0.025	0.004	T	C	0.057	33.595
Body mass index (BMI)	rs1229984	100239319	4.60E-10	4	0.037	0.006	C	T	0.973	38.845
Body mass index (BMI)	rs12364470	134601012	4.90E-13	11	0.019	0.003	G	T	0.165	52.255
Body mass index (BMI)	rs12440603	46585722	3.40E-12	15	0.014	0.002	T	C	0.434	48.447
Body mass index (BMI)	rs12459368	18459377	2.50E-14	19	-0.017	0.002	G	A	0.268	58.089
Body mass index (BMI)	rs12462975	30272202	2.60E-20	19	0.020	0.002	A	G	0.330	85.263
Body mass index (BMI)	rs12507026	45181334	6.30E-48	4	0.029	0.002	T	A	0.435	211.556
Body mass index (BMI)	rs12541408	95585807	1.60E-11	8	-0.014	0.002	C	T	0.317	45.355
Body mass index (BMI)	rs1266874	51779638	1.00E-11	6	0.014	0.002	G	A	0.350	46.322
Body mass index (BMI)	rs12681792	62054463	3.50E-09	8	0.015	0.003	A	C	0.193	34.901
Body mass index (BMI)	rs12692596	161265910	1.30E-10	2	0.013	0.002	T	C	0.372	41.262
Body mass index (BMI)	rs12696039	156304750	3.50E-08	3	-0.015	0.003	G	A	0.149	30.419
Body mass index (BMI)	rs1286058	91458523	6.20E-12	14	0.015	0.002	A	T	0.704	47.261
Body mass index (BMI)	rs12881629	101146413	7.70E-10	14	0.022	0.004	G	A	0.083	37.825
Body mass index (BMI)	rs12889639	103860206	7.70E-14	14	0.016	0.002	A	G	0.651	55.893
Body mass index (BMI)	rs12921986	72312727	3.80E-08	16	0.020	0.004	G	A	0.078	30.226
Body mass index (BMI)	rs12937411	34950239	1.40E-17	17	-0.017	0.002	T	C	0.408	72.837
Body mass index (BMI)	rs1296328	137083193	3.90E-21	4	-0.019	0.002	C	A	0.559	89.033
Body mass index (BMI)	rs12974458	1866115	2.30E-14	19	0.015	0.002	T	C	0.543	58.280
Body mass index (BMI)	rs13012070	35447243	6.10E-09	2	-0.014	0.002	A	G	0.228	33.798
Body mass index (BMI)	rs13033310	133523605	3.30E-08	2	0.013	0.002	A	G	0.253	30.524
Body mass index (BMI)	rs13097918	35676330	1.70E-09	3	0.015	0.002	A	T	0.212	36.296
Body mass index (BMI)	rs13107325	103188709	8.50E-37	4	0.048	0.004	T	C	0.075	160.574
Body mass index (BMI)	rs13176429	43152216	3.10E-11	5	0.014	0.002	C	T	0.688	44.116
Body mass index (BMI)	rs1320251	21264396	1.50E-19	17	-0.018	0.002	T	C	0.455	81.773
Body mass index (BMI)	rs13218383	120173501	5.80E-12	6	-0.014	0.002	G	C	0.335	47.381
Body mass index (BMI)	rs1322842	20488897	1.10E-10	6	-0.013	0.002	G	A	0.609	41.608
Body mass index (BMI)	rs13248187	14336834	2.00E-12	8	0.016	0.002	C	T	0.269	49.458
Body mass index (BMI)	rs1327259	51177811	2.80E-13	6	-0.015	0.002	G	A	0.388	53.345
Body mass index (BMI)	rs13291723	80510077	3.40E-08	9	0.011	0.002	A	G	0.571	30.463
Body mass index (BMI)	rs1330199	27760946	3.30E-09	9	-0.012	0.002	T	G	0.483	34.995
Body mass index (BMI)	rs13420048	50751414	4.60E-14	2	-0.015	0.002	A	C	0.365	56.882
Body mass index (BMI)	rs13427822	213414265	5.60E-16	2	-0.018	0.002	G	A	0.271	65.581
Body mass index (BMI)	rs1346841	65651730	9.70E-11	4	-0.013	0.002	A	G	0.405	41.891
Body mass index (BMI)	rs1360201	73796450	4.80E-11	9	0.013	0.002	T	C	0.482	43.268
Body mass index (BMI)	rs13642	30432220	4.30E-15	11	-0.016	0.002	T	A	0.361	61.540
Body mass index (BMI)	rs140159717	73765586	2.80E-11	15	-0.025	0.004	T	C	0.082	44.290
Body mass index (BMI)	rs1438945	152510937	1.10E-09	5	-0.013	0.002	A	T	0.715	37.072
Body mass index (BMI)	rs1441264	79580919	3.40E-18	13	0.018	0.002	A	G	0.594	75.639
Body mass index (BMI)	rs1451963	41350367	7.40E-10	14	0.022	0.004	T	G	0.082	37.908
Body mass index (BMI)	rs1454687	94038085	6.10E-26	3	-0.021	0.002	G	C	0.515	110.943
Body mass index (BMI)	rs1458156	41887940	1.20E-12	12	0.014	0.002	T	C	0.488	50.557
Body mass index (BMI)	rs145981104	74714869	2.00E-08	8	0.023	0.004	G	A	0.064	31.515
Body mass index (BMI)	rs146569428	2199686	2.00E-08	11	0.014	0.002	A	G	0.201	31.510
Body mass index (BMI)	rs1471093	108031094	4.10E-11	3	0.013	0.002	A	G	0.617	43.548
Body mass index (BMI)	rs1471740	136328270	8.90E-18	3	0.019	0.002	C	T	0.741	73.753
Body mass index (BMI)	rs147568678	93061851	1.10E-08	10	-0.013	0.002	C	T	0.238	32.734
Body mass index (BMI)	rs1477290	87988934	2.20E-31	5	0.034	0.003	C	T	0.137	135.833
Body mass index (BMI)	rs147730268	123024476	1.30E-22	12	-0.035	0.004	T	G	0.087	95.826
Body mass index (BMI)	rs1503526	63020706	5.90E-15	5	0.015	0.002	C	T	0.480	60.932
Body mass index (BMI)	rs156201	104847441	8.30E-09	6	0.013	0.002	C	G	0.753	33.195
Body mass index (BMI)	rs156914	16848652	1.60E-08	1	0.011	0.002	A	G	0.492	31.964
Body mass index (BMI)	rs1582931	122657199	2.30E-11	5	-0.013	0.002	A	G	0.473	44.698
Body mass index (BMI)	rs159037	94197477	4.90E-08	5	0.012	0.002	C	T	0.254	29.761
Body mass index (BMI)	rs1608113	157815217	9.30E-09	3	-0.012	0.002	T	A	0.365	32.974
Body mass index (BMI)	rs1609010	77227464	7.90E-26	8	0.021	0.002	G	A	0.566	110.420
Body mass index (BMI)	rs1657930	57120989	3.50E-09	15	-0.015	0.002	A	G	0.803	34.888
Body mass index (BMI)	rs16916303	30823761	4.20E-10	9	-0.019	0.003	G	A	0.120	39.027
Body mass index (BMI)	rs17056301	158271680	2.20E-09	5	0.014	0.002	C	T	0.256	35.815
Body mass index (BMI)	rs17132130	2108036	7.50E-14	7	-0.018	0.002	C	G	0.221	55.933
Body mass index (BMI)	rs17149254	76634463	7.00E-17	7	-0.021	0.003	C	T	0.805	69.664
Body mass index (BMI)	rs17218879	14768540	1.60E-09	9	0.013	0.002	G	C	0.339	36.369
Body mass index (BMI)	rs17289010	140774684	1.60E-10	4	-0.013	0.002	G	A	0.328	40.948
Body mass index (BMI)	rs17399739	87490850	4.40E-12	10	0.027	0.004	G	A	0.069	47.935

Body mass index (BMI)	rs17446299	40762556	8.90E-09	13	0.015	0.003	G	C	0.166	33.058
Body mass index (BMI)	rs17544384	115295160	5.30E-09	1	0.014	0.002	C	T	0.211	34.073
Body mass index (BMI)	rs17668356	61208619	1.50E-16	3	-0.023	0.003	G	C	0.146	68.125
Body mass index (BMI)	rs17770336	28414625	1.30E-30	9	0.024	0.002	T	C	0.322	132.354
Body mass index (BMI)	rs1778830	156489974	7.50E-12	1	0.014	0.002	A	G	0.362	46.884
Body mass index (BMI)	rs1788808	21090023	7.70E-25	18	-0.020	0.002	G	A	0.495	105.921
Body mass index (BMI)	rs1793636	131934926	4.70E-10	11	-0.013	0.002	C	G	0.309	38.796
Body mass index (BMI)	rs1805123	150645534	3.00E-13	7	-0.017	0.002	G	T	0.245	53.214
Body mass index (BMI)	rs1834144	40744790	8.60E-12	18	-0.014	0.002	A	C	0.373	46.617
Body mass index (BMI)	rs1860750	73309738	3.50E-09	14	0.012	0.002	A	T	0.511	34.872
Body mass index (BMI)	rs1861410	58933591	1.20E-26	2	-0.021	0.002	T	C	0.555	114.184
Body mass index (BMI)	rs1884897	6612832	2.30E-22	20	0.020	0.002	G	A	0.627	94.605
Body mass index (BMI)	rs1919243	88778861	5.50E-09	5	0.012	0.002	C	T	0.487	34.022
Body mass index (BMI)	rs1967772	28036062	1.00E-14	13	-0.017	0.002	A	G	0.285	59.811
Body mass index (BMI)	rs2035936	141298124	1.70E-17	3	0.037	0.004	T	G	0.056	72.426
Body mass index (BMI)	rs2051559	3298800	2.80E-12	4	0.020	0.003	C	T	0.133	48.858
Body mass index (BMI)	rs2075466	4872970	2.80E-09	16	0.013	0.002	C	G	0.267	35.320
Body mass index (BMI)	rs2102278	52818664	2.00E-08	4	0.012	0.002	G	A	0.322	31.468
Body mass index (BMI)	rs2133561	139086651	5.80E-12	5	-0.014	0.002	T	A	0.611	47.406
Body mass index (BMI)	rs213518	26941065	1.80E-08	7	0.016	0.003	C	T	0.146	31.688
Body mass index (BMI)	rs2153740	2126089	1.60E-08	20	-0.011	0.002	G	A	0.480	31.888
Body mass index (BMI)	rs215634	32369148	2.40E-14	7	-0.016	0.002	G	A	0.612	58.186
Body mass index (BMI)	rs2172131	133978962	9.00E-14	10	-0.015	0.002	C	T	0.579	55.573
Body mass index (BMI)	rs217672	62361021	2.40E-14	14	0.017	0.002	C	A	0.272	58.197
Body mass index (BMI)	rs2192158	55505360	3.70E-14	4	-0.015	0.002	G	A	0.553	57.309
Body mass index (BMI)	rs2216931	181599070	5.20E-16	2	0.017	0.002	A	C	0.662	65.729
Body mass index (BMI)	rs2234458	65639374	3.60E-23	11	-0.020	0.002	T	C	0.640	98.316
Body mass index (BMI)	rs2248551	131924689	3.80E-08	6	0.015	0.003	A	G	0.165	30.229
Body mass index (BMI)	rs2253310	108888593	2.10E-17	6	0.017	0.002	G	C	0.626	72.013
Body mass index (BMI)	rs2271189	56494991	6.50E-16	12	-0.016	0.002	A	G	0.403	65.267
Body mass index (BMI)	rs2289379	44804225	5.20E-14	7	-0.015	0.002	T	C	0.396	56.658
Body mass index (BMI)	rs2307111	75003678	1.30E-43	5	-0.028	0.002	C	T	0.395	191.810
Body mass index (BMI)	rs2342892	24540806	1.30E-10	16	-0.013	0.002	G	T	0.516	41.242
Body mass index (BMI)	rs2381404	144035442	1.30E-09	2	0.014	0.002	C	T	0.244	36.882
Body mass index (BMI)	rs2383377	33257914	3.90E-08	14	0.016	0.003	A	G	0.131	30.185
Body mass index (BMI)	rs2396625	113028634	5.60E-21	7	-0.019	0.002	A	T	0.421	88.299
Body mass index (BMI)	rs2398861	96430747	2.10E-15	9	0.018	0.002	G	A	0.259	62.978
Body mass index (BMI)	rs2425816	44895075	1.20E-09	20	0.012	0.002	A	G	0.415	36.973
Body mass index (BMI)	rs2433733	230816703	3.80E-16	2	-0.017	0.002	A	G	0.678	66.357
Body mass index (BMI)	rs2439823	99778226	5.30E-22	10	0.019	0.002	G	A	0.546	92.986
Body mass index (BMI)	rs2482356	94178371	1.30E-08	9	-0.011	0.002	C	T	0.429	32.372
Body mass index (BMI)	rs2512892	131451862	9.20E-11	11	0.013	0.002	C	T	0.566	41.981
Body mass index (BMI)	rs252761	77380723	1.20E-08	5	-0.012	0.002	T	G	0.588	32.511
Body mass index (BMI)	rs2568958	72765116	1.60E-28	1	0.022	0.002	A	G	0.604	122.760
Body mass index (BMI)	rs2569993	12926096	2.40E-09	3	0.013	0.002	C	T	0.320	35.650
Body mass index (BMI)	rs2606228	183537759	2.70E-11	3	-0.014	0.002	C	A	0.646	44.362
Body mass index (BMI)	rs2616143	20632022	6.70E-11	8	-0.014	0.002	A	G	0.320	42.607
Body mass index (BMI)	rs2618039	112324111	1.40E-12	1	0.014	0.002	T	A	0.381	50.209
Body mass index (BMI)	rs2678204	201800511	3.80E-31	1	0.024	0.002	G	T	0.340	134.724
Body mass index (BMI)	rs2725371	30854033	1.10E-13	8	-0.016	0.002	G	A	0.696	55.155
Body mass index (BMI)	rs2791643	11207269	7.20E-09	1	-0.013	0.002	T	C	0.762	33.472
Body mass index (BMI)	rs2814942	34644261	5.30E-42	6	0.028	0.002	A	G	0.330	184.405
Body mass index (BMI)	rs28350	42418446	2.90E-12	3	-0.018	0.003	G	A	0.821	48.768
Body mass index (BMI)	rs28366156	31671498	1.60E-19	6	-0.026	0.003	C	T	0.131	81.705
Body mass index (BMI)	rs2837996	42626706	1.10E-09	21	0.013	0.002	C	T	0.651	37.124
Body mass index (BMI)	rs28404639	80874229	1.20E-08	5	-0.012	0.002	T	C	0.366	32.476
Body mass index (BMI)	rs28489620	41804716	2.80E-12	22	-0.015	0.002	A	G	0.290	48.822
Body mass index (BMI)	rs28568418	53462969	1.20E-08	15	-0.018	0.003	A	G	0.108	32.477
Body mass index (BMI)	rs2861685	67837553	1.00E-17	2	-0.017	0.002	C	T	0.412	73.511
Body mass index (BMI)	rs2870111	79403585	7.10E-15	15	-0.016	0.002	T	C	0.412	60.577
Body mass index (BMI)	rs2875762	124925032	3.30E-11	6	0.015	0.002	C	G	0.243	44.013
Body mass index (BMI)	rs2899644	59470366	2.30E-10	15	0.015	0.002	T	C	0.230	40.184
Body mass index (BMI)	rs2920503	12324230	1.70E-10	3	-0.014	0.002	T	C	0.285	40.797
Body mass index (BMI)	rs2922757	76544012	1.30E-09	8	0.012	0.002	T	A	0.597	36.782
Body mass index (BMI)	rs2962334	86879056	7.80E-10	5	0.043	0.007	T	G	0.020	37.800
Body mass index (BMI)	rs317656	69681101	6.10E-11	12	-0.014	0.002	A	T	0.724	42.790
Body mass index (BMI)	rs3213943	136389840	4.80E-10	2	-0.018	0.003	A	C	0.132	38.761
Body mass index (BMI)	rs32421	167362416	2.60E-08	5	0.013	0.002	T	A	0.224	30.971
Body mass index (BMI)	rs329118	133861663	1.30E-16	5	-0.017	0.002	T	C	0.419	68.409

Body mass index (BMI)	rs329651	133767622	3.30E-10	11	0.016	0.003	T	G	0.804	39.484
Body mass index (BMI)	rs34045288	40369081	3.50E-29	6	0.023	0.002	T	C	0.334	125.749
Body mass index (BMI)	rs34153025	41339697	9.60E-09	15	-0.039	0.007	C	T	0.022	32.929
Body mass index (BMI)	rs34234296	175166636	2.40E-13	2	-0.015	0.002	A	G	0.392	53.668
Body mass index (BMI)	rs34481751	47501038	7.80E-12	20	-0.019	0.003	A	C	0.165	46.822
Body mass index (BMI)	rs34517439	78450517	3.60E-37	1	0.039	0.003	A	C	0.122	162.251
Body mass index (BMI)	rs34696181	93096635	8.10E-09	7	0.011	0.002	C	T	0.476	33.255
Body mass index (BMI)	rs347551	119389031	5.40E-12	5	0.014	0.002	G	C	0.472	47.542
Body mass index (BMI)	rs34811474	25408838	4.10E-34	4	-0.029	0.002	A	G	0.231	148.304
Body mass index (BMI)	rs349071	84776849	1.90E-11	11	-0.013	0.002	A	G	0.500	45.057
Body mass index (BMI)	rs35154326	24862414	5.30E-09	16	-0.013	0.002	G	A	0.274	34.073
Body mass index (BMI)	rs35364449	74278126	9.20E-12	15	0.022	0.003	T	C	0.110	46.497
Body mass index (BMI)	rs355777	154034950	3.30E-14	3	0.015	0.002	C	G	0.408	57.546
Body mass index (BMI)	rs35697587	47298505	9.20E-17	14	-0.016	0.002	A	G	0.508	69.126
Body mass index (BMI)	rs35697691	52353498	6.20E-11	15	0.023	0.004	G	C	0.089	42.753
Body mass index (BMI)	rs35809007	47019521	9.10E-17	2	-0.017	0.002	A	G	0.363	69.164
Body mass index (BMI)	rs35957544	73440371	1.10E-22	8	-0.020	0.002	T	G	0.574	96.040
Body mass index (BMI)	rs36007635	163009335	2.20E-13	6	-0.021	0.003	A	G	0.138	53.849
Body mass index (BMI)	rs36061954	38329650	2.00E-10	8	0.013	0.002	T	C	0.399	40.461
Body mass index (BMI)	rs3764625	49649051	5.20E-09	19	-0.012	0.002	G	T	0.588	34.120
Body mass index (BMI)	rs3784710	68072458	2.50E-36	15	-0.030	0.002	C	T	0.227	158.391
Body mass index (BMI)	rs3803286	103246470	6.40E-19	14	-0.019	0.002	G	A	0.667	78.930
Body mass index (BMI)	rs3807566	50564204	1.50E-09	7	-0.012	0.002	T	G	0.438	36.590
Body mass index (BMI)	rs3814883	29994922	1.00E-33	16	0.024	0.002	T	C	0.482	146.427
Body mass index (BMI)	rs3845344	75001480	5.40E-16	1	0.016	0.002	T	C	0.391	65.649
Body mass index (BMI)	rs3851998	131876605	2.00E-09	3	-0.014	0.002	G	C	0.743	35.972
Body mass index (BMI)	rs3866805	6657424	1.20E-08	1	0.012	0.002	A	C	0.356	32.547
Body mass index (BMI)	rs3897102	123492112	2.50E-09	12	0.012	0.002	T	C	0.411	35.533
Body mass index (BMI)	rs3901286	99107727	2.70E-16	7	-0.023	0.003	A	C	0.152	66.978
Body mass index (BMI)	rs3902951	69789755	2.00E-09	14	0.014	0.002	G	T	0.237	35.928
Body mass index (BMI)	rs3935190	79084367	4.00E-13	17	-0.014	0.002	A	G	0.537	52.621
Body mass index (BMI)	rs394608	46581798	9.50E-21	21	0.019	0.002	C	T	0.538	87.268
Body mass index (BMI)	rs396755	104022239	6.30E-10	5	-0.012	0.002	G	C	0.571	38.241
Body mass index (BMI)	rs40071	107496102	3.80E-24	5	-0.026	0.003	C	T	0.180	102.732
Body mass index (BMI)	rs4017425	44028764	2.10E-10	3	-0.013	0.002	T	C	0.470	40.364
Body mass index (BMI)	rs4055791	59266053	7.40E-19	13	-0.018	0.002	T	C	0.417	78.652
Body mass index (BMI)	rs406388	18226997	8.30E-10	22	0.016	0.003	G	C	0.177	37.690
Body mass index (BMI)	rs41279738	110082551	4.00E-28	1	0.068	0.006	G	T	0.026	120.925
Body mass index (BMI)	rs4148155	89054667	1.40E-13	4	-0.023	0.003	G	A	0.113	54.662
Body mass index (BMI)	rs4261944	31003636	1.60E-11	4	0.014	0.002	G	T	0.365	45.391
Body mass index (BMI)	rs4267103	60966740	1.40E-09	12	0.015	0.003	C	T	0.186	36.630
Body mass index (BMI)	rs4284600	31843528	2.00E-09	15	0.012	0.002	C	T	0.467	35.982
Body mass index (BMI)	rs429343	147903382	3.30E-18	2	-0.017	0.002	G	A	0.577	75.717
Body mass index (BMI)	rs429358	45411941	2.40E-22	19	-0.027	0.003	C	T	0.154	94.501
Body mass index (BMI)	rs4307239	24354300	1.00E-09	7	0.012	0.002	G	A	0.459	37.293
Body mass index (BMI)	rs4419475	96150044	1.10E-08	4	0.011	0.002	T	A	0.407	32.641
Body mass index (BMI)	rs4444317	92573234	2.50E-11	15	-0.016	0.002	G	A	0.216	44.555
Body mass index (BMI)	rs4456769	25190777	3.60E-12	20	0.015	0.002	T	C	0.333	48.346
Body mass index (BMI)	rs4477562	54104968	2.90E-23	13	0.030	0.003	T	C	0.129	98.734
Body mass index (BMI)	rs4482463	205375909	3.00E-17	2	-0.031	0.004	A	C	0.923	71.375
Body mass index (BMI)	rs45486197	2244849	1.70E-10	19	0.026	0.004	A	G	0.066	40.728
Body mass index (BMI)	rs4605363	229010960	3.30E-15	2	0.016	0.002	C	A	0.342	62.052
Body mass index (BMI)	rs4648450	2723214	8.40E-14	1	-0.015	0.002	A	C	0.467	55.715
Body mass index (BMI)	rs4658403	243832560	1.00E-12	1	-0.019	0.003	T	C	0.834	50.801
Body mass index (BMI)	rs4672338	60217457	8.00E-11	2	0.014	0.002	T	C	0.336	42.257
Body mass index (BMI)	rs4722398	3125220	7.90E-11	7	0.019	0.003	T	C	0.136	42.290
Body mass index (BMI)	rs4737188	64756657	3.50E-10	8	-0.012	0.002	T	A	0.474	39.395
Body mass index (BMI)	rs4764949	103658096	3.10E-18	12	-0.018	0.002	G	A	0.326	75.841
Body mass index (BMI)	rs4790292	1824305	2.60E-20	17	-0.025	0.003	A	C	0.154	85.278
Body mass index (BMI)	rs4820410	40690385	1.70E-17	22	-0.018	0.002	G	A	0.345	72.421
Body mass index (BMI)	rs4832298	86764004	5.40E-14	2	-0.016	0.002	T	C	0.686	56.568
Body mass index (BMI)	rs4858940	88254820	1.40E-13	3	0.023	0.003	C	T	0.886	54.712
Body mass index (BMI)	rs4876611	116671848	3.30E-19	8	0.020	0.002	G	A	0.720	80.265
Body mass index (BMI)	rs4929923	8639200	4.50E-20	11	0.019	0.002	C	T	0.645	84.197
Body mass index (BMI)	rs5011579	69187318	1.60E-10	16	0.014	0.002	G	C	0.715	40.903
Body mass index (BMI)	rs512121	7548501	2.60E-10	18	-0.016	0.003	C	T	0.192	39.934
Body mass index (BMI)	rs529200	173114305	1.30E-17	3	0.017	0.002	G	A	0.528	72.933
Body mass index (BMI)	rs539515	177889025	2.00E-91	1	0.050	0.002	C	A	0.205	411.165
Body mass index (BMI)	rs55707359	46159333	6.20E-11	11	0.053	0.008	G	T	0.015	42.771

Body mass index (BMI)	rs55714539	18207397	6.00E-17	19	0.018	0.002	C	A	0.344	69.988
Body mass index (BMI)	rs55726687	991306	1.40E-24	12	0.025	0.002	A	G	0.210	104.730
Body mass index (BMI)	rs55769038	13331808	1.20E-15	11	0.016	0.002	A	G	0.590	64.067
Body mass index (BMI)	rs558887	28712741	1.50E-09	11	-0.013	0.002	G	A	0.308	36.587
Body mass index (BMI)	rs559231	39644247	3.40E-11	18	0.013	0.002	T	G	0.393	43.921
Body mass index (BMI)	rs55966114	97584357	6.50E-09	12	0.015	0.003	T	C	0.193	33.689
Body mass index (BMI)	rs56038322	69925128	9.00E-11	3	0.014	0.002	A	G	0.311	42.029
Body mass index (BMI)	rs56094641	53806453	1.00E-200	16	0.073	0.002	G	A	0.405	######
Body mass index (BMI)	rs56133507	172818467	2.70E-08	2	0.014	0.002	G	T	0.197	30.948
Body mass index (BMI)	rs56143236	157020444	2.30E-08	3	0.013	0.002	T	C	0.256	31.265
Body mass index (BMI)	rs56161855	46288649	1.40E-14	17	0.022	0.003	T	A	0.133	59.238
Body mass index (BMI)	rs56203622	131040874	1.40E-10	9	0.018	0.003	C	T	0.146	41.167
Body mass index (BMI)	rs56352336	19352155	2.90E-09	19	-0.016	0.003	C	T	0.155	35.244
Body mass index (BMI)	rs56399737	33381721	6.40E-16	13	-0.016	0.002	T	C	0.449	65.295
Body mass index (BMI)	rs56858768	86511730	2.40E-13	13	0.016	0.002	A	G	0.297	53.628
Body mass index (BMI)	rs56893062	25662655	6.20E-09	8	0.013	0.002	G	T	0.303	33.758
Body mass index (BMI)	rs56930105	10982487	3.70E-08	2	0.016	0.003	T	C	0.139	30.309
Body mass index (BMI)	rs57636386	58048295	1.10E-30	18	-0.041	0.004	C	T	0.084	132.557
Body mass index (BMI)	rs57989773	100629078	1.60E-08	6	0.013	0.002	C	T	0.245	31.908
Body mass index (BMI)	rs58862095	75081418	2.40E-30	7	-0.023	0.002	T	C	0.419	131.076
Body mass index (BMI)	rs59068084	113256737	3.80E-08	4	0.011	0.002	T	G	0.410	30.237
Body mass index (BMI)	rs59086897	25145173	2.00E-64	2	0.033	0.002	A	T	0.488	287.231
Body mass index (BMI)	rs59227842	43692423	1.50E-26	11	0.023	0.002	G	A	0.311	113.786
Body mass index (BMI)	rs594024	69443822	1.70E-13	11	-0.015	0.002	C	T	0.554	54.353
Body mass index (BMI)	rs6023655	53479658	3.60E-10	20	-0.015	0.002	G	A	0.766	39.314
Body mass index (BMI)	rs60764613	1839911	1.10E-13	18	0.021	0.003	T	G	0.145	55.133
Body mass index (BMI)	rs61740466	19934900	5.60E-09	1	-0.014	0.002	A	G	0.237	33.971
Body mass index (BMI)	rs61813324	156049877	2.80E-23	1	0.029	0.003	T	C	0.136	98.785
Body mass index (BMI)	rs61828641	174321997	1.20E-12	1	0.022	0.003	A	G	0.109	50.539
Body mass index (BMI)	rs61871615	102487140	1.00E-13	10	-0.027	0.004	T	C	0.092	55.297
Body mass index (BMI)	rs61903695	89922417	2.50E-13	11	0.017	0.002	G	A	0.255	53.574
Body mass index (BMI)	rs62007782	78029797	9.40E-14	15	-0.017	0.002	A	G	0.265	55.480
Body mass index (BMI)	rs62020775	89960286	7.20E-09	15	-0.017	0.003	A	T	0.142	33.471
Body mass index (BMI)	rs62072006	52938468	3.00E-08	17	0.016	0.003	C	A	0.145	30.714
Body mass index (BMI)	rs62107261	422144	4.60E-87	2	-0.091	0.005	C	T	0.048	391.192
Body mass index (BMI)	rs62176243	166190881	6.10E-11	2	-0.015	0.002	T	A	0.245	42.779
Body mass index (BMI)	rs62190049	182566998	4.00E-08	2	-0.011	0.002	C	G	0.390	30.148
Body mass index (BMI)	rs62241847	20466465	5.30E-09	3	-0.012	0.002	G	A	0.314	34.068
Body mass index (BMI)	rs62246311	9498143	1.80E-10	3	0.021	0.003	A	G	0.102	40.657
Body mass index (BMI)	rs62379271	105870033	5.00E-09	5	0.012	0.002	G	T	0.579	34.171
Body mass index (BMI)	rs62407562	33530346	8.50E-11	6	0.014	0.002	A	T	0.269	42.129
Body mass index (BMI)	rs6265	27679916	3.30E-56	11	-0.040	0.003	T	C	0.188	249.501
Body mass index (BMI)	rs6430068	145627927	5.30E-09	2	0.019	0.003	A	G	0.109	34.060
Body mass index (BMI)	rs6444950	170602073	8.30E-12	3	0.016	0.002	A	G	0.237	46.687
Body mass index (BMI)	rs6545714	59307725	2.40E-24	2	-0.021	0.002	A	G	0.601	103.650
Body mass index (BMI)	rs6560906	133414054	1.20E-08	12	-0.012	0.002	C	T	0.692	32.449
Body mass index (BMI)	rs6561937	58257667	4.60E-12	13	-0.016	0.002	A	T	0.754	47.851
Body mass index (BMI)	rs6567160	57829135	2.30E-118	18	0.054	0.002	C	T	0.233	534.974
Body mass index (BMI)	rs6575340	94023972	8.70E-24	14	0.021	0.002	A	G	0.636	101.113
Body mass index (BMI)	rs6597975	838842	7.10E-12	11	0.014	0.002	G	C	0.544	47.012
Body mass index (BMI)	rs66679256	18351898	6.90E-14	4	0.015	0.002	T	C	0.446	56.095
Body mass index (BMI)	rs6669341	47678458	1.60E-17	1	-0.017	0.002	G	A	0.583	72.596
Body mass index (BMI)	rs6682438	33784146	3.70E-10	1	0.013	0.002	C	T	0.673	39.245
Body mass index (BMI)	rs6705567	55320173	9.80E-13	2	-0.015	0.002	C	T	0.376	50.879
Body mass index (BMI)	rs6707827	100123030	3.90E-08	2	0.012	0.002	G	A	0.704	30.202
Body mass index (BMI)	rs6710091	239597	1.60E-08	2	-0.012	0.002	G	C	0.348	31.947
Body mass index (BMI)	rs6713781	40291940	2.20E-11	2	-0.014	0.002	C	G	0.402	44.798
Body mass index (BMI)	rs6725931	220205146	3.30E-12	2	0.019	0.003	T	C	0.848	48.473
Body mass index (BMI)	rs6744646	628504	4.50E-100	2	0.055	0.003	G	A	0.828	450.922
Body mass index (BMI)	rs6752979	81741750	3.30E-09	2	0.013	0.002	A	G	0.317	34.988
Body mass index (BMI)	rs67609008	126640936	8.60E-15	10	0.017	0.002	C	T	0.284	60.204
Body mass index (BMI)	rs6769617	62687746	8.10E-11	3	-0.014	0.002	T	A	0.664	42.230
Body mass index (BMI)	rs6774894	196116393	9.70E-11	3	0.013	0.002	A	T	0.358	41.872
Body mass index (BMI)	rs6777784	62376645	1.00E-08	3	0.012	0.002	T	G	0.617	32.803
Body mass index (BMI)	rs6831088	20257769	2.20E-08	4	-0.012	0.002	A	G	0.640	31.312
Body mass index (BMI)	rs6843852	162132758	3.50E-11	4	0.013	0.002	T	C	0.508	43.879
Body mass index (BMI)	rs6909685	97753952	4.70E-12	6	-0.015	0.002	T	C	0.327	47.798
Body mass index (BMI)	rs6922607	142703483	3.20E-09	6	0.015	0.003	G	A	0.190	35.033
Body mass index (BMI)	rs6938973	98421721	1.80E-19	6	0.018	0.002	C	T	0.601	81.481

Body mass index (BMI)	rs6950388	1270699	2.30E-10	7	0.016	0.002	A	G	0.795	40.202
Body mass index (BMI)	rs6962980	113452183	1.00E-15	7	-0.016	0.002	C	A	0.556	64.417
Body mass index (BMI)	rs698147	3513485	9.60E-11	5	-0.013	0.002	G	A	0.544	41.909
Body mass index (BMI)	rs7024334	109072075	6.70E-09	9	-0.014	0.002	G	T	0.779	33.611
Body mass index (BMI)	rs7027304	129408290	3.10E-12	9	0.015	0.002	T	C	0.653	48.598
Body mass index (BMI)	rs7034554	37081301	2.10E-10	9	-0.013	0.002	G	A	0.374	40.401
Body mass index (BMI)	rs7038943	120377178	1.80E-11	9	-0.014	0.002	C	T	0.339	45.155
Body mass index (BMI)	rs704061	89771903	1.70E-13	12	0.015	0.002	C	T	0.455	54.347
Body mass index (BMI)	rs705158	125232995	1.30E-11	10	0.016	0.002	A	T	0.245	45.798
Body mass index (BMI)	rs7070670	61842645	6.00E-09	10	-0.012	0.002	T	C	0.328	33.835
Body mass index (BMI)	rs7081254	132955696	6.20E-09	10	-0.014	0.002	C	T	0.206	33.778
Body mass index (BMI)	rs7124681	47529947	1.50E-37	11	0.026	0.002	A	C	0.408	164.058
Body mass index (BMI)	rs7132908	50263148	1.40E-48	12	0.030	0.002	A	G	0.384	214.590
Body mass index (BMI)	rs71495038	33971383	6.70E-14	10	0.028	0.004	A	G	0.077	56.158
Body mass index (BMI)	rs7201895	407723	6.00E-13	16	-0.015	0.002	A	G	0.354	51.855
Body mass index (BMI)	rs7206608	82872628	1.80E-10	16	0.014	0.002	G	C	0.322	40.702
Body mass index (BMI)	rs7218014	65832016	2.90E-14	17	0.019	0.002	C	T	0.197	57.833
Body mass index (BMI)	rs7232171	31251221	8.00E-10	18	0.012	0.002	T	G	0.583	37.761
Body mass index (BMI)	rs723672	2161561	3.00E-08	12	0.011	0.002	T	C	0.432	30.739
Body mass index (BMI)	rs7250833	33937277	6.70E-10	19	0.014	0.002	T	C	0.289	38.092
Body mass index (BMI)	rs7259070	47562509	6.60E-27	19	0.022	0.002	C	T	0.596	115.341
Body mass index (BMI)	rs72634826	1601052	1.00E-20	1	-0.021	0.002	A	G	0.260	87.086
Body mass index (BMI)	rs72649373	80609966	6.10E-10	4	0.018	0.003	C	T	0.143	38.278
Body mass index (BMI)	rs72673947	118884379	9.90E-12	8	0.022	0.003	G	A	0.107	46.344
Body mass index (BMI)	rs72892910	50816887	1.50E-49	6	0.039	0.003	T	G	0.172	218.994
Body mass index (BMI)	rs72976986	4050424	7.60E-20	19	-0.023	0.003	A	G	0.190	83.146
Body mass index (BMI)	rs73026725	31017686	4.90E-16	19	-0.022	0.003	A	C	0.154	65.839
Body mass index (BMI)	rs73052033	185828465	7.70E-33	3	-0.030	0.003	C	T	0.185	142.466
Body mass index (BMI)	rs7306534	68107914	4.10E-08	12	-0.011	0.002	A	G	0.622	30.124
Body mass index (BMI)	rs73124396	71579606	3.10E-10	7	-0.015	0.002	C	T	0.205	39.597
Body mass index (BMI)	rs73142879	51195932	3.60E-26	20	-0.027	0.003	T	C	0.192	111.977
Body mass index (BMI)	rs73193736	108294381	2.20E-14	12	-0.018	0.002	G	A	0.244	58.314
Body mass index (BMI)	rs73213484	28489339	1.70E-15	4	-0.023	0.003	T	A	0.141	63.355
Body mass index (BMI)	rs7331420	99236471	6.60E-11	13	-0.014	0.002	A	G	0.285	42.634
Body mass index (BMI)	rs7357754	92207308	1.10E-12	9	0.014	0.002	G	A	0.500	50.631
Body mass index (BMI)	rs73601548	18549889	1.30E-08	10	0.018	0.003	T	C	0.115	32.388
Body mass index (BMI)	rs73985439	212299249	1.70E-10	2	0.014	0.002	C	A	0.307	40.732
Body mass index (BMI)	rs7442885	87682877	3.20E-21	5	-0.023	0.002	G	C	0.214	89.444
Body mass index (BMI)	rs745249	105460333	1.10E-15	2	0.018	0.002	T	C	0.282	64.247
Body mass index (BMI)	rs74750282	114744463	2.50E-08	7	-0.020	0.004	C	T	0.087	31.027
Body mass index (BMI)	rs7498665	28883241	2.30E-40	16	0.027	0.002	G	A	0.400	176.877
Body mass index (BMI)	rs7516554	210301331	2.60E-09	1	0.012	0.002	T	C	0.400	35.482
Body mass index (BMI)	rs7519259	66434743	1.70E-12	1	0.014	0.002	A	G	0.528	49.823
Body mass index (BMI)	rs7539903	209208033	8.30E-11	1	-0.013	0.002	A	T	0.616	42.196
Body mass index (BMI)	rs754635	42305131	1.50E-12	3	0.022	0.003	G	C	0.887	50.029
Body mass index (BMI)	rs75499503	26145217	8.90E-14	6	-0.018	0.002	T	C	0.220	55.599
Body mass index (BMI)	rs7568228	236848488	1.30E-09	2	-0.012	0.002	C	G	0.527	36.766
Body mass index (BMI)	rs7571496	6169351	1.80E-12	2	-0.016	0.002	G	A	0.261	49.684
Body mass index (BMI)	rs76183894	114371939	1.70E-09	3	-0.022	0.004	C	T	0.081	36.304
Body mass index (BMI)	rs7619139	25110415	2.20E-11	3	0.013	0.002	A	T	0.589	44.774
Body mass index (BMI)	rs765874	143185557	1.00E-09	6	-0.012	0.002	A	T	0.489	37.256
Body mass index (BMI)	rs76702514	195148296	1.20E-11	1	-0.016	0.002	G	C	0.211	45.924
Body mass index (BMI)	rs7683836	180167906	8.20E-10	4	-0.012	0.002	A	G	0.557	37.712
Body mass index (BMI)	rs7704382	112448114	1.80E-09	5	0.012	0.002	G	C	0.434	36.209
Body mass index (BMI)	rs7707394	74472939	8.60E-21	5	-0.019	0.002	A	G	0.357	87.463
Body mass index (BMI)	rs7708584	153543466	1.40E-15	5	-0.016	0.002	G	A	0.572	63.783
Body mass index (BMI)	rs7761673	70357368	1.30E-08	6	-0.014	0.002	A	T	0.220	32.337
Body mass index (BMI)	rs7762794	153380228	9.10E-12	6	0.015	0.002	G	A	0.285	46.505
Body mass index (BMI)	rs7774	4801163	3.30E-12	17	0.015	0.002	A	C	0.310	48.521
Body mass index (BMI)	rs7776021	73742152	1.40E-08	6	0.012	0.002	A	G	0.288	32.124
Body mass index (BMI)	rs7802342	137435925	1.80E-08	7	0.012	0.002	G	T	0.289	31.662
Body mass index (BMI)	rs7805441	78121458	1.80E-11	7	0.013	0.002	T	C	0.502	45.224
Body mass index (BMI)	rs78086698	24024639	3.60E-10	12	0.032	0.005	C	T	0.040	39.334
Body mass index (BMI)	rs784257	53397199	2.00E-12	18	0.018	0.003	C	T	0.813	49.461
Body mass index (BMI)	rs78605811	83631491	1.80E-13	3	-0.033	0.004	C	A	0.054	54.200
Body mass index (BMI)	rs7893571	16750129	2.30E-11	10	0.014	0.002	T	G	0.666	44.731
Body mass index (BMI)	rs7924036	65191645	5.10E-13	10	-0.014	0.002	T	G	0.503	52.157
Body mass index (BMI)	rs7925100	118941596	3.30E-13	11	0.015	0.002	A	G	0.396	53.028
Body mass index (BMI)	rs7944782	130795698	2.20E-15	11	0.016	0.002	G	T	0.510	62.884

Body mass index (BMI)	rs7947143	64090422	8.30E-12	11	-0.018	0.003	A	G	0.163	46.682
Body mass index (BMI)	rs79780963	104952499	1.50E-10	10	0.024	0.004	T	C	0.077	41.052
Body mass index (BMI)	rs7996639	97019090	3.60E-13	13	0.015	0.002	A	G	0.449	52.839
Body mass index (BMI)	rs80135274	5286277	3.80E-08	17	0.021	0.004	T	A	0.070	30.230
Body mass index (BMI)	rs8015400	25930988	6.70E-24	14	0.021	0.002	A	C	0.677	101.625
Body mass index (BMI)	rs8020365	79937216	1.00E-25	14	0.025	0.002	A	T	0.220	109.878
Body mass index (BMI)	rs8024137	35837297	1.60E-08	15	0.016	0.003	T	A	0.848	31.902
Body mass index (BMI)	rs8025516	95271872	1.70E-12	15	-0.015	0.002	G	T	0.646	49.849
Body mass index (BMI)	rs8076669	15888448	1.80E-12	17	0.014	0.002	C	T	0.562	49.676
Body mass index (BMI)	rs8089514	69224478	3.90E-10	18	0.013	0.002	A	T	0.369	39.145
Body mass index (BMI)	rs8112818	18812785	1.80E-24	19	-0.021	0.002	G	A	0.400	104.235
Body mass index (BMI)	rs8132491	40288577	2.40E-12	21	-0.015	0.002	A	G	0.313	49.146
Body mass index (BMI)	rs815163	190294726	1.00E-16	1	-0.016	0.002	C	T	0.563	68.913
Body mass index (BMI)	rs852042	17091233	1.40E-08	20	-0.013	0.002	G	A	0.759	32.176
Body mass index (BMI)	rs862320	69651866	1.20E-30	16	-0.023	0.002	T	C	0.410	132.429
Body mass index (BMI)	rs879620	4015729	2.30E-32	16	0.024	0.002	T	C	0.613	140.278
Body mass index (BMI)	rs909001	32196647	8.00E-10	1	0.016	0.003	G	C	0.172	37.759
Body mass index (BMI)	rs909892	41982698	2.60E-10	20	-0.018	0.003	A	G	0.135	39.956
Body mass index (BMI)	rs923994	67802992	1.30E-09	4	-0.015	0.002	G	A	0.783	36.819
Body mass index (BMI)	rs9267671	31880480	2.60E-10	6	0.026	0.004	A	G	0.061	39.974
Body mass index (BMI)	rs9291822	64076515	8.60E-13	5	-0.014	0.002	T	C	0.515	51.132
Body mass index (BMI)	rs9294260	83433228	1.00E-13	6	0.015	0.002	A	G	0.477	55.278
Body mass index (BMI)	rs9349235	42516718	2.60E-08	6	0.011	0.002	T	C	0.411	30.966
Body mass index (BMI)	rs935166	26949366	3.20E-16	2	-0.016	0.002	A	G	0.507	66.648
Body mass index (BMI)	rs9388446	126064920	2.00E-08	6	0.011	0.002	A	T	0.516	31.461
Body mass index (BMI)	rs9463175	9510030	4.40E-08	6	-0.012	0.002	T	C	0.339	29.956
Body mass index (BMI)	rs9478496	154333183	1.10E-11	6	0.018	0.003	C	T	0.164	46.210
Body mass index (BMI)	rs9515446	112217108	3.20E-14	13	0.015	0.002	G	A	0.448	57.586
Body mass index (BMI)	rs9522180	111970212	1.50E-12	13	-0.014	0.002	T	C	0.553	50.107
Body mass index (BMI)	rs9571687	67472713	1.40E-10	13	-0.014	0.002	A	C	0.329	41.195
Body mass index (BMI)	rs961498	107713511	1.80E-09	12	0.012	0.002	C	G	0.503	36.126
Body mass index (BMI)	rs9638713	14645949	1.20E-08	7	-0.036	0.006	G	A	0.975	32.411
Body mass index (BMI)	rs9673839	76895693	5.50E-11	16	0.013	0.002	G	A	0.491	42.991
Body mass index (BMI)	rs9830592	104631603	1.00E-14	3	0.015	0.002	A	C	0.582	59.847
Body mass index (BMI)	rs9835772	85766025	4.70E-13	3	0.017	0.002	T	A	0.244	52.328
Body mass index (BMI)	rs9839081	123051230	4.60E-08	3	-0.012	0.002	A	G	0.325	29.884
Body mass index (BMI)	rs9843653	49920571	2.90E-50	3	0.029	0.002	C	T	0.512	222.278
Body mass index (BMI)	rs9876664	85806313	9.60E-19	3	-0.018	0.002	T	G	0.375	78.135
Body mass index (BMI)	rs9888533	107854612	2.70E-09	13	0.012	0.002	T	C	0.538	35.376
Body mass index (BMI)	rs9926784	19941968	8.60-21	16	-0.024	0.003	C	T	0.185	87.464
Body mass index (BMI)	rs9951619	56882326	9.50E-10	18	0.014	0.002	G	T	0.767	37.421
