# Supplementary material for: The relationship between obstructive sleep apnea and osteoarthritis: evidence from an observational and Mendelian randomization study
Source: Front Neurol. 2024 Jun 28;15:1425327. doi: 10.3389/fneur.2024.1425327 (PMC11239388; doi:10.3389/fneur.2024.1425327)
Supplement: Supplementary file 6 [file Table_5.pdf]

mediation	exposure	outcome	method	nsnp	OR(95%CI)	pval
Waist-hip ratio	OSA	OA	MR Egger	92	1.12 (0.97 to 1.30)	0.138
			Weighted median	92	1.12 (1.00 to 1.26)	0.049
			Inverse variance weighted	92	1.13 (1.05 to 1.21)	0.001
			Simple mode	92	1.19 (0.90 to 1.56)	0.225
			Weighted mode	92	1.10 (0.95 to 1.27)	0.22
	Waist-hip ratio	OA	MR Egger	306	1.06 (0.79 to 1.42)	0.71
			Weighted median	306	0.97 (0.81 to 1.15)	0.701
			Inverse variance weighted	306	0.94 (0.83 to 1.07)	0.351
			Simple mode	306	1.02 (0.66 to 1.58)	0.929
			Weighted mode	306	0.97 (0.74 to 1.28)	0.842
	OSA	Waist-hip ratio	MR Egger	98	0.98 (0.95 to 1.02)	0.291
			Weighted median	98	1.00 (0.98 to 1.01)	0.802
			Inverse variance weighted	98	0.99 (0.97 to 1.01)	0.381
			Simple mode	98	1.02 (0.99 to 1.06)	0.204
			Weighted mode	98	1.00 (0.98 to 1.02)	0.916
Waist-to-hip ratio adjusted for BMI	OSA	OA	MR Egger	92	1.12 (0.97 to 1.30)	0.138
			Weighted median	92	1.12 (1.00 to 1.26)	0.049
			Inverse variance weighted	92	1.13 (1.05 to 1.21)	0.001
			Simple mode	92	1.19 (0.90 to 1.56)	0.225
			Weighted mode	92	1.10 (0.95 to 1.27)	0.22
	Waist-to-hip ratio adjusted for BMI	OA	MR Egger	234	0.86 (0.61 to 1.21)	0.392
			Weighted median	234	1.05 (0.86 to 1.28)	0.642
			Inverse variance weighted	234	1.04 (0.91 to 1.20)	0.549
			Simple mode	234	1.29 (0.77 to 2.15)	0.333
			Weighted mode	234	1.12 (0.81 to 1.55)	0.504
	OSA	Waist-to-hip ratio adjusted for BMI	MR Egger	29	0.96 (0.86 to 1.07)	0.439
			Weighted median	29	0.99 (0.96 to 1.02)	0.547
			Inverse variance weighted	29	0.98 (0.95 to 1.01)	0.184
			Simple mode	29	1.03 (0.98 to 1.07)	0.241
			Weighted mode	29	1.00 (0.96 to 1.03)	0.854
Waist circumference	OSA	OA	MR Egger	92	1.12 (0.97 to 1.30)	0.138
			Weighted median	92	1.12 (1.00 to 1.26)	0.049
			Inverse variance weighted	92	1.13 (1.05 to 1.21)	0.001
			Simple mode	92	1.19 (0.90 to 1.56)	0.225
			Weighted mode	92	1.10 (0.95 to 1.27)	0.22
	Waist circumference	OA	MR Egger	263	1.42 (0.97 to 2.07)	0.072
			Weighted median	263	1.71 (1.37 to 2.13)	0
			Inverse variance weighted	263	1.63 (1.42 to 1.86)	0
			Simple mode	263	1.50 (0.81 to 2.76)	0.194
			Weighted mode	263	1.81 (1.21 to 2.71)	0.004
	OSA	Waist circumference (UKB data field [illegible]	MR Egger	85	1.03 (0.96 to 1.10)	0.396
			Weighted median	85	1.02 (1.00 to 1.04)	0.116
			Inverse variance weighted	85	1.09 (1.05 to 1.13)	0
			Simple mode	85	1.03 (0.99 to 1.07)	0.112
			Weighted mode	85	1.01 (0.99 to 1.03)	0.234
Modified Stumvoll Insulin Sensitivity Index	OSA	OA	MR Egger	92	1.12 (0.97 to 1.30)	0.138
			Weighted median	92	1.12 (1.00 to 1.26)	0.049
			Inverse variance weighted	92	1.13 (1.05 to 1.21)	0.001
			Simple mode	92	1.19 (0.90 to 1.56)	0.225
			Weighted mode	92	1.10 (0.95 to 1.27)	0.22
	Modified Stumvoll Insulin Sensitivity [illegible]	OA	MR Egger	8	1.07 (0.54 to 2.11)	0.856
			Weighted median	8	1.08 (0.83 to 1.41)	0.546
			Inverse variance weighted	8	1.07 (0.82 to 1.39)	0.631
			Simple mode	8	1.09 (0.72 to 1.67)	0.695
			Weighted mode	8	1.09 (0.75 to 1.59)	0.661
	OSA	Modified Stumvoll Insulin Sensitivity [illegible]	MR Egger	44	0.92 (0.68 to 1.24)	0.586
			Weighted median	44	0.86 (0.76 to 0.97)	0.016
			Inverse variance weighted	44	0.90 (0.83 to 0.97)	0.01
			Simple mode	44	0.78 (0.59 to 1.02)	0.081
			Weighted mode	44	0.82 (0.66 to 1.01)	0.068
Modified Stumvoll Insulin Sensitivity Index (model [illegible]	OSA	OA	MR Egger	92	1.12 (0.97 to 1.30)	0.138
			Weighted median	92	1.12 (1.00 to 1.26)	0.049
			Inverse variance weighted	92	1.13 (1.05 to 1.21)	0.001
			Simple mode	92	1.19 (0.90 to 1.56)	0.225
			Weighted mode	92	1.10 (0.95 to 1.27)	0.22
	Modified Stumvoll Insulin Sensitivity [illegible]	OA	MR Egger	8	1.16 (0.69 to 1.95)	0.601
			Weighted median	8	1.03 (0.82 to 1.30)	0.796
			Inverse variance weighted	8	0.94 (0.78 to 1.13)	0.518
			Simple mode	8	1.06 (0.73 to 1.52)	0.781
			Weighted mode	8	1.06 (0.78 to 1.43)	0.74
		Modified [illegible]	MR Egger	44	1.15 (0.84 to 1.59)	0.388

adjusted for BMI)	OSA	Stumvoll Insulin Sensitivity Index (model adjusted	Weighted median	44	1.02 (0.92 to 1.14)	0.668
			Inverse variance weighted	44	1.00 (0.92 to 1.09)	0.962
			Simple mode	44	1.07 (0.81 to 1.42)	0.638
			Weighted mode	44	1.12 (0.91 to 1.38)	0.3
Homeostasis model assessment of insulin resistance	OSA	OA	MR Egger	92	1.12 (0.97 to 1.30)	0.138
			Weighted median	92	1.12 (1.00 to 1.26)	0.049
			Inverse variance weighted	92	1.13 (1.05 to 1.21)	0.001
			Simple mode	92	1.19 (0.90 to 1.56)	0.225
			Weighted mode	92	1.10 (0.95 to 1.27)	0.22
	Homeostasis model assessment of insulin	OA	MR Egger	12	0.30 (0.07 to 1.21)	0.121
			Weighted median	12	0.75 (0.39 to 1.42)	0.372
			Inverse variance weighted	12	0.87 (0.54 to 1.39)	0.555
			Simple mode	12	0.83 (0.29 to 2.39)	0.742
			Weighted mode	12	0.68 (0.26 to 1.76)	0.445
	OSA	Homeostasis model assessment of insulin	MR Egger	53	1.17 (1.02 to 1.34)	0.031
			Weighted median	53	1.02 (0.98 to 1.07)	0.262
			Inverse variance weighted	53	1.05 (1.01 to 1.09)	0.007
			Simple mode	53	0.97 (0.88 to 1.09)	0.642
			Weighted mode	53	0.98 (0.88 to 1.10)	0.751
Hip circumference	OSA	OA	MR Egger	92	1.12 (0.97 to 1.30)	0.138
			Weighted median	92	1.12 (1.00 to 1.26)	0.049
			Inverse variance weighted	92	1.13 (1.05 to 1.21)	0.001
			Simple mode	92	1.19 (0.90 to 1.56)	0.225
			Weighted mode	92	1.10 (0.95 to 1.27)	0.22
	Hip circumference	OA	MR Egger	312	1.76 (1.23 to 2.52)	0.002
			Weighted median	312	1.39 (1.18 to 1.64)	0
			Inverse variance weighted	312	1.39 (1.21 to 1.59)	0
			Simple mode	312	1.42 (0.85 to 2.35)	0.182
			Weighted mode	312	1.50 (1.02 to 2.22)	0.04
	OSA	Hip circumference	MR Egger	85	1.04 (0.96 to 1.12)	0.347
			Weighted median	85	1.02 (1.00 to 1.04)	0.132
			Inverse variance weighted	85	1.11 (1.07 to 1.15)	0
			Simple mode	85	1.03 (0.98 to 1.07)	0.215
			Weighted mode	85	1.01 (0.99 to 1.03)	0.208
Fasting insulin	OSA	OA	MR Egger	92	1.12 (0.97 to 1.30)	0.138
			Weighted median	92	1.12 (1.00 to 1.26)	0.049
			Inverse variance weighted	92	1.13 (1.05 to 1.21)	0.001
			Simple mode	92	1.19 (0.90 to 1.56)	0.225
			Weighted mode	92	1.10 (0.95 to 1.27)	0.22
	Fasting insul	OA	MR Egger	106	0.74 (0.34 to 1.65)	0.466
			Weighted median	106	0.97 (0.65 to 1.44)	0.869
			Inverse variance weighted	106	0.98 (0.73 to 1.30)	0.874
			Simple mode	106	0.84 (0.33 to 2.15)	0.713
			Weighted mode	106	1.16 (0.50 to 2.70)	0.736
	OSA	Fasting insulin	MR Egger	100	0.98 (0.96 to 1.01)	0.273
			Weighted median	100	1.00 (0.98 to 1.02)	0.849
			Inverse variance weighted	100	0.99 (0.98 to 1.00)	0.126
			Simple mode	100	1.01 (0.97 to 1.06)	0.527
			Weighted mode	100	1.00 (0.98 to 1.03)	0.89
BMI	OSA	OA	MR Egger	92	1.12 (0.97 to 1.30)	0.138
			Weighted median	92	1.12 (1.00 to 1.26)	0.049
			Inverse variance weighted	92	1.13 (1.05 to 1.21)	0.001
			Simple mode	92	1.19 (0.90 to 1.56)	0.225
			Weighted mode	92	1.10 (0.95 to 1.27)	0.22
	Body mass index	OA	MR Egger	437	1.45 (1.11 to 1.89)	0.007
			Weighted median	437	1.62 (1.38 to 1.91)	0
			Inverse variance weighted	437	1.49 (1.35 to 1.64)	0
			Simple mode	437	1.09 (0.67 to 1.77)	0.727
			Weighted mode	437	1.79 (1.34 to 2.39)	0
	OSA	Body mass index (BMI)	MR Egger	97	1.06 (0.98 to 1.15)	0.141
			Weighted median	97	1.03 (1.01 to 1.05)	0.012
			Inverse variance weighted	97	1.12 (1.08 to 1.17)	0
			Simple mode	97	1.05 (1.01 to 1.09)	0.011
			Weighted mode	97	1.03 (1.00 to 1.05)	0.029
